# Patient characteristics associated with retrospectively self-reported treatment outcomes following psychological therapy for anxiety or depressive disorders - a cohort of GLAD study participants

**DOI:** 10.1186/s12888-022-04275-6

**Published:** 2022-11-18

**Authors:** Christopher Rayner, Jonathan R.I. Coleman, Megan Skelton, Cherie Armour, John Bradley, Joshua E.J. Buckman, Molly R. Davies, Colette R. Hirsch, Matthew Hotopf, Christopher Hübel, Ian R. Jones, Gursharan Kalsi, Nathalie Kingston, Georgina Krebs, Yuhao Lin, Dina Monssen, Andrew M. McIntosh, Jessica R. Mundy, Alicia J. Peel, Katharine A. Rimes, Henry C. Rogers, Daniel J. Smith, Abigail R. ter Kuile, Katherine N. Thompson, David Veale, Janet Wingrove, James T.R. Walters, Gerome Breen, Thalia C. Eley

**Affiliations:** 1 Institute of Psychiatry, Psychology & Neuroscience, King’s College London, London, UK; 2 UK National Institute for Health and Care Research (NIHR) Biomedical Research Centre, South London and Maudsley NHS Trust, London, UK; 3 Research Centre for Stress Trauma & Related Conditions (STARC), School of Psychology, Queen’s University Belfast (QUB), Belfast, Northern Ireland UK; 4 NIHR BioResource, Cambridge University Hospitals NHS Foundation, Cambridge Biomedical Campus, Cambridge, UK; 5 Centre for Outcomes Research and Effectiveness (CORE), Research Department of Clinical, Educational & Health Psychology, University College London, 1-19 Torrington Place, WC1E 7HB London, UK; 6 iCope – Camden & Islington Psychological Therapies Services – Camden & Islington NHS Foundation Trust, St Pancras Hospital, NW1 0PE London, UK; 7 South London and Maudsley NHS Foundation Trust, Denmark Hill, SE5 8AZ London, UK; 8 Aarhus Business and Social Sciences, National Centre for Register-based Research, Aarhus University, Aarhus, Denmark; 9 MRC Centre for Neuropsychiatric Genetics and Genomics, Division of Psychological Medicine and Clinical Neurosciences, School of Medicine, Cardiff University, Cardiff, UK; 10 Department of Haematology, University of Cambridge, Cambridge Biomedical Campus, Cambridge, UK; 11 Division of Psychiatry, Centre for Clinical Brain Sciences, University of Edinburgh, Edinburgh, UK; 12 Institute of Health and Wellbeing, University of Glasgow, Glasgow, UK

**Keywords:** Cognitive behavioral therapy, Counselling, Minimal phenotyping

## Abstract

**Background:**

Progress towards stratified care for anxiety and depression will require the identification of new predictors. We collected data on retrospectively self-reported therapeutic outcomes in adults who received psychological therapy in the UK in the past ten years. We aimed to replicate factors associated with traditional treatment outcome measures from the literature.

**Methods:**

Participants were from the Genetic Links to Anxiety and Depression (GLAD) Study, a UK-based volunteer cohort study. We investigated associations between retrospectively self-reported outcomes following therapy, on a five-point scale (global rating of change; GRC) and a range of sociodemographic, clinical and therapy-related factors, using ordinal logistic regression models (*n* = 2890).

**Results:**

Four factors were associated with therapy outcomes (adjusted odds ratios, OR). One sociodemographic factor, having university-level education, was associated with favourable outcomes (OR = 1.37, 95%CI: 1.18, 1.59). Two clinical factors, greater number of reported episodes of illness (OR = 0.95, 95%CI: 0.92, 0.97) and higher levels of personality disorder symptoms (OR = 0.89, 95%CI: 0.87, 0.91), were associated with less favourable outcomes. Finally, reported regular use of additional therapeutic activities was associated with favourable outcomes (OR = 1.39, 95%CI: 1.19, 1.63). There were no statistically significant differences between fully adjusted multivariable and unadjusted univariable odds ratios.

**Conclusion:**

Therapy outcome data can be collected quickly and inexpensively using retrospectively self-reported measures in large observational cohorts. Retrospectively self-reported therapy outcomes were associated with four factors previously reported in the literature. Similar data collected in larger observational cohorts may enable detection of novel associations with therapy outcomes, to generate new hypotheses, which can be followed up in prospective studies.

**Supplementary Information:**

The online version contains supplementary material available at 10.1186/s12888-022-04275-6.

## Introduction

Anxiety and depressive disorders account for more than 70 million years lived with disability globally each year [1]. Several effective treatments exist, but outcomes following psychological therapy are variable and many individuals do not experience improvement [2–5]. Development of a statistical model that predicts the likelihood of success following each available treatment, for each patient, on their first assessment is a major research goal. Identifying clinical, sociodemographic and genetic predictors of treatment outcome could facilitate this goal. Well-replicated associations with poor outcomes following treatment include clinical factors related to symptom severity, chronicity and comorbidity, such as pre-treatment symptom severity [6, 7], duration of illness [8–10], age of onset [11], history of previous treatments for major depressive disorder [12], functional impairment [13, 14] and comorbid anxiety disorders [15], dysthymia [16] or personality disorder [17–20]. Associations with sociodemographic factors have been less consistent. In large meta-analyses, no effects of age or sex on therapy outcomes were observed [11]. Lower cognitive ability [12] and social support [11], and stressful life events have been associated with unfavourable treatment outcomes [11]. Furthermore, lower levels of patient engagement and poor therapeutic alliance is associated with less favourable outcomes [6, 21].

Despite evidence for many associated factors, few statistical models can prospectively predict mental health treatment outcomes outside of the settings used to develop the models [22, 23]. A common explanation for the inconsistent associations across studies is low statistical power due to aetiological heterogeneity, small sample sizes and small effect sizes [23]. Efforts to increase statistical power have typically focused on either increasing sample size, or restricting analyses to more homogeneous subgroups. Cohort studies often collect detailed social, demographic and clinical data and may provide opportunities for further data linkage with national registries [24]. As such, observational cohorts may provide increased sample sizes for testing associations across a broad range of variables with outcomes following therapy. While such associations may only have small individual effects, they may eventually be combined with a range of factors to produce clinically meaningful prediction models. Furthermore, larger samples could allow for analyses to be stratified into more homogeneous subgroups and for interactions between predictive factors to be tested [23]. Linkage between cohort studies and medical records is the optimal strategy to achieve this aim, however, there are various challenges associated with data linkage [25]. Supplementary approaches such as retrospective data collection in existing cohorts may also help to provide the boost in sample size necessary for analyses of therapy outcomes. For example, some cohorts have made use of a single, self-report item to measure depression, which has been described as a minimal-phenotyping approach [26, 27]. In a UK Biobank study, a single item: “self-reported past treatment-seeking for problems with nerves, anxiety, tension or depression”, was used as a “broad depression” measure [28]. This measure increased the number of “depression cases” from 8,276 to 113,769 and 14 novel genetic associations were discovered [28]. While minimal phenotyping will likely measure somewhat different constructs from clinical assessments [26], the benefit of such strategies is that data can be collected on a larger scale, faster and at a substantially lower cost than clinically ascertained outcome measurements [23, 27]. Thus, such approaches should not replace traditional methods of measurement but can be used in large population based cohorts to collect data on therapy outcomes [23]. Exploratory analyses in observational cohorts could be used to generate new hypotheses about new prognostic factors that can subsequently be tested in prospective clinical studies. However, it will also be necessary to assess the validity of phenotypes derived from minimal approaches, by comparison with gold-standard approaches.

We used the Global Rating of Change (GRC) to retrospectively measure patient-perceived outcomes following psychological therapy in a large observational cohort. The patient-rated GRC has high test retest reliability [29], high face validity [30] and has been used in research to calculate the minimal clinically important change on symptom questionnaires [31]. For example, the minimum change in depression symptom scores (measured using the Patient Health Questionnaire 9-item version; PHQ-9 [32]) that was associated with reporting feeling “better” on the GRC was a reduction of ~ 1.7 points (~ 21%) [33]. We aimed to replicate associations from the literature between psychological therapy outcomes measured using clinical scales and sociodemographic, clinical and therapy related factors. Based on the literature, we hypothesised that individuals reporting less favourable outcomes would have higher symptom severity, chronicity and comorbidity (more episodes, earlier age of onset, more comorbid diagnoses, higher personality disorder symptom scores) and individuals reporting more favourable outcomes would have higher educational attainment. Given that evidence is less clear for other sociodemographic factors, we did not hypothesise about the effects of age, sex or ethnicity. Additional therapy-related factors were drawn from the therapy questionnaire and were included as covariates rather than primary analysis variables. We sought to demonstrate the utility of minimal phenotyping to generate a large sample for detecting expected associations with treatment outcomes.

## Methods

### Participants

Participants were from the Genetic Links to Anxiety and Depression (GLAD) Study, an online study of UK residents recruited via a nationwide advertising campaign and through NHS services [24]. Participants were recruited if they met criteria for a lifetime diagnosis of an anxiety or depressive disorder based on the Composite International Diagnostic Interview Short Form (CIDI-SF) [34, 35]. At GLAD sign-up, data were collected on demographics, mental and physical health symptoms and disorders, and a range of psychological and behavioural phenotypes relevant to anxiety and depression [24]. Following sign-up, participants are re-contactable and have the opportunity to participate in additional research studies and phases of data collection. At the time of this analysis, there were 37,413 participants who had completed the GLAD sign-up questionnaire (GSU-Q) between 09/2018 and 10/2020 (79.6% female; aged: 16–80 years, mean = 39, SD = 14).

Between 08/2019 and 10/2020, GLAD participants were invited to take part in a therapy history and outcomes questionnaire (THO-Q) (S. Figure 2). Analyses were restricted to respondents who had received psychological therapy to treat major depressive disorder (MDD), generalized anxiety disorder (GAD), specific phobia (SpP), social phobia (SoP) or panic disorder (PD). Those who reported treatment for other primary psychiatric diagnoses (e.g. personality disorder, bipolar disorder) were excluded from analyses. Psychological treatment included **one-to-one** cognitive-behavioral therapy (CBT), counseling or other one-to-one therapy and **group** CBT, counseling, or other group therapy. Those who reported use of *other* one-to-one or group therapy types were excluded from analyses, because these were mostly non-traditional therapies (i.e. those not typically offered in primary care; e.g. acceptance and commitment therapy, eye movement desensitization and reprocessing). We further restricted analyses to individuals who were at least 18 years old during their therapy and who had received their most recent course of therapy within the last ten years (2010–2020). This timeframe was chosen to increase likelihood of reliable recall and also to account for likely changes in therapy protocols and guidelines.

### Measures

#### Outcome: global rating of change following the most recent course of therapy

Self-rated change in symptoms following therapy was retrospectively reported by participants on the therapy history and outcomes questionnaire. Participants were asked how much their symptoms and day-to-day functioning had improved following their course of talking therapy. There were five response options for this outcome, which was taken from the Global Rating of Change (GRC; [31]): much worse (-2), a little worse (-1), no change (0), a little better (+ 1), much better (+ 2; Table 1).

#### Analysis variables: sociodemographic, clinical and therapy-related factors.

We aimed to cover a breadth of factors for which there is some evidence, taking an inclusive approach to ensure a broad base of factors were covered. The participants in this study reported on their *history* of therapy and outcomes *retrospectively.* Therefore, we selected only core sociodemographic and clinical factors that reflected their overall course of illness, and therapy factors that related to the specific time that they were receiving their therapy.

Sociodemographic variables were mostly collected during the sign-up questionnaire. For our analysis, these included self-reported biological sex (male/female), ethnic background and education level. Given that the majority of the sample (95.3%) reported their ethnicity to be White British, we dichotomised ethnicity into two groups for comparison: (1) white British and (2) a collective UK ethnic minority group. Education level was dichotomised from six categorical responses, using the median category (University degree: yes/no). Age (at time of therapy in years), was retrospectively reported at THO-Q assessment.

Four clinical factors were included in analyses: (1) self-reported age of onset of the first episode of either depression or anxiety disorder, (2) the number of episodes of illness, (3) a personality disorder symptom score measured using the Standardized Assessment of Severity of Personality Disorder (SASPD [36]), and (4) number of psychiatric comorbidities during therapy. The first three factors were assessed at entry into the study and the fourth was assessed in the therapy history questionnaire.

Several ‘therapy factors’ derived from the therapy history questionnaire were included as explanatory variables or as covariates. Y*ears since therapy* was calculated as self-reported age at the time of treatment subtracted from age at time of completing the questionnaire. *Primary diagnosis* was self-reported MDD, GAD or a combined group PPD. *Type of therapy* was either one-to-one CBT, one-to-one counseling or group therapy. Given that participants who received any group therapy were a minority, we combined both group CBT and group counseling into one category for analysis. *Concurrent medications* asked whether the participant was taking antidepressant or anti-anxiety medications during the course of therapy (yes/no). *First-time therapy* was whether the participant had only ever received therapy once (yes/no). *Use of therapeutic activities* described whether the participant reported using an activity (e.g. yoga, mindfulness or meditation) as a form of self-help (yes/no).

### Statistical analysis

#### Regression analyses

Univariable and multivariable ordinal logistic regression models were fitted using maximum likelihood estimation using the lrm function from the rms package [37] in R version 3.6.1 [38]. Effect sizes estimated from multivariable and univariable models were compared using two-sample z-tests (S.Table 12). A Brant test [39] was performed to assess the assumption of proportionality in the proportional odds model (S.Table 10). A Bonferroni p-value was calculated to correct for multiple effectively independent tests, which was computed as the number of principal components that explained 99.5% of the variance in the correlation matrix of all explanatory variables (S.Table 9; S. Figure 3; see: [40, 41]). The correlation matrix of explanatory variables also showed that none of the variables selected for the regression analyses were strongly correlated (all absolute r < 0.37). Post-hoc variance inflation factors (VIF) were also computed to assess multicollinearity in the multivariable regression models, using the rms package [37]. The maximum VIF calculated across all analyses was for *age of onset* (VIF = 1.29, i.e. an inflation of 29%, which is moderate but not considered as problematic; S.Table 11).

#### Sensitivity analyses

Prior to analysis, the characteristics of participants with complete data were compared with participants with missing data (S.Tables 1 and 3). To reduce the impact of missing data on our findings, “missing data” variables were dummy coded and included in analyses so that all participants were retained for analysis. For categorical variables, a “missing data” category was coded to replace missing values. Continuous variables were mean imputed and “missing data” variables were dummy coded and included as covariates. This approach was taken because data were unlikely to be missing at random and thus multiple imputation was not appropriate. As a sensitivity analysis, effect sizes from models with missing data indicators were compared with those estimated in complete-case analyses (S.Table 13).

We also tested whether time-varying confounding had an impact on our results. Participants were stratified into three groups. Group 1 included participants who received their most recent course of therapy before completing the GSU-Q. Group 2 included participants who were receiving therapy approximately concurrently to completing the GSU-Q. Group 3 included participants who received therapy after signing up and completing the sign-up questionnaire. Results are presented in S.Table 7 and S. Figure 4. Effect size estimates were compared across strata and with the primary analysis using two-sample z-tests (S.Table 14). Finally, to assess the impact of length of time between THO-Q completion and time of therapy, analyses were repeated using a more restrictive cut-off (5 years) and a less restrictive cut-off (15 years) compared the primary analysis (10 year cut-off). Effect size estimates were compared using two-sample z-tests (S.Table 15).

## Results

### Characteristics of the analysis sample

At the time of analysis 4814 GLAD participants (12.9% of 37,413; S. Figure 1) responded to the therapy history and outcomes questionnaire and 4380 respondents reported receiving psychological therapy at least once (90.1% of THO-Q respondents). Within these therapy receivers (79.8% female), 632 (14.4%) had received one course of therapy and 3727 (85.6%) had received more than one course of therapy in their lifetime. All subsequent analyses related to each participant’s most recent course of therapy. There were 2890 GLAD participants who reported receiving psychological therapy for a disorder of interest as an adult (i.e. aged 18 + at the time of receiving therapy), in the previous ten years (2010–2020). Further details on all analysis variables are provided in Table 1 and S.Tables 1, 2 and 3.

**Table 1:** Analysis variables in a subsample of the Genetic Links to Anxiety and Depression (GLAD) study participants (n = 2890) who received psychological therapy (cognitive behavioural therapy or counseling) for major depressive disorder, generalised anxiety disorder, or phobic/panic disorders

	Female (n = 2284)	Male (n = 606)	Total (N = 2890)
**Self-reported change**			
Much worse	59 (2.6%)	11 (1.8%)	70 (2.4%)
A little worse	85 (3.7%)	28 (4.6%)	113 (3.9%)
No change	465 (20.4%)	161 (26.6%)	626 (21.7%)
A little better	970 (42.5%)	246 (40.6%)	1216 (42.1%)
Much better	705 (30.9%)	160 (26.4%)	865 (29.9%)
**Age at signup**			
Mean (SD)	38.1 (13.5)	43.8 (13.5)	39.3 (13.7)
Range	16.4–79.9	17.0–77.9	16.4–79.9
**Age during therapy**			
Mean (SD)	37.1 (13.2)	42.5 (13.2)	38.3 (13.4)
Range	18.0–79.0	18.0–78.0	18.0–79.0
**Ethnicity**			
Arab	2 (0.1%)	0 (0.0%)	2 (0.1%)
Asian or asian british	18 (0.8%)	9 (1.5%)	27 (0.9%)
Black or black british	8 (0.4%)	1 (0.2%)	9 (0.3%)
Mixed	64 (2.8%)	7 (1.2%)	71 (2.5%)
Other	16 (0.7%)	10 (1.7%)	26 (0.9%)
White	2172 (95.3%)	578 (95.5%)	2750 (95.3%)
Missing data	4	1	5
**University degree**			
No	804 (35.8%)	232 (39.1%)	1036 (36.5%)
Yes	1443 (64.2%)	361 (60.9%)	1804 (63.5%)
Missing data	37	13	50
**Age of onset**			
Mean (SD)	18.0 (8.7)	22.1 (11.6)	18.9 (9.5)
Range	5.0–61.0	5.0–61.0	5.0–61.0
Missing data	136	59	195
**Number of episodes**			
Mean (SD)	9.0 (3.7)	9.4 (3.7)	9.1 (3.7)
Range	1.0–13.0	1.0–13.0	1.0–13.0
Missing data	548	147	695
**Number of comorbidities**			
Mean (SD)	1.4 (1.3)	1.3 (1.3)	1.4 (1.3)
Range	0.0–6.0	0.0–5.0	0.0–6.0
**Personality disorder score**			
Mean (SD)	8.1 (3.5)	8.0 (3.6)	8.1 (3.5)
Range	0.0–23.0	0.0–23.0	0.0–23.0
Missing data	1	0	1
**First therapy**			
No	1989 (87.5%)	510 (84.4%)	2499 (86.9%)
Yes	284 (12.5%)	94 (15.6%)	378 (13.1%)
Missing data	11	2	13
**Years since therapy at followup**			
Mean (SD)	2.4 (2.4)	2.8 (2.7)	2.5 (2.5)
Range	0.0–10.0	0.0–10.0	0.0–10.0
**Main diagnosis**			
Major depressive disorder	1329 (58.2%)	382 (63.0%)	1711 (59.2%)
Generalised anxiety disorder	754 (33.0%)	169 (27.9%)	923 (31.9%)
Phobia (various)	42 (1.8%)	0 (0.0%)	42 (1.5%)
Social anxiety disorder	102 (4.5%)	40 (6.6%)	142 (4.9%)
Panic disorder	57 (2.5%)	15 (2.5%)	72 (2.5%)
**Therapy type**			
One-to-one CBT	949 (41.5%)	262 (43.2%)	1211 (41.9%)
One-to-one counselling	1199 (52.5%)	284 (46.9%)	1483 (51.3%)
Group CBT	114 (5.0%)	48 (7.9%)	162 (5.6%)
Group counselling	22 (1.0%)	12 (2.0%)	34 (1.2%)
**Concurrent medications**			
No	514 (22.6%)	151 (25.3%)	665 (23.2%)
Yes	1759 (77.4%)	447 (74.7%)	2206 (76.8%)
Missing data	11	8	19
**Regular therapeutic activity**			
No	572 (25.9%)	218 (37.7%)	790 (28.3%)
Yes	1636 (74.1%)	361 (62.3%)	1997 (71.7%)
Missing data	76	27	103

### Factors associated with retrospectively self-reported therapy outcomes

For a summary of our findings see Table 2. Overall, the findings for univariable and multivariable analyses were consistent. Brant tests showed that the assumption of proportionality in the proportional odds model was not violated (Omnibus test: p = 0.31; S.Table 10). Factors associated with less favourable outcomes were: number of episodes, personality disorder symptoms, being male, and receiving therapy for the first time (versus having had a previous course of therapy). Factors associated with favourable outcomes were: having university-level education, and use of an additional therapeutic activity. After adjustment for all other factors and multiple testing, only four factors had statistically significant associations: more illness episodes and greater personality disorder symptom severity were associated with poor outcomes, and higher educational attainment and reported regular use of a therapeutic activity were associated with favourable outcomes.

**Table 2:** Summary statistics from multivariable (MV) and univariable (UV) proportional odds ordinal logistic regression models using maximum likelihood estimation to test for associations between self-rated therapy outcomes (global rating of improvement) and sociodemographic, clinical and therapy factors self-reported in a subsample of the Genetic Links to Anxiety and Depression (GLAD) study participants (n = 2890) who received psychological therapy (cognitive behavioural therapy or counseling) for major depressive disorder, generalised anxiety disorder, or phobic/panic disorders

Variable	OR_UV_	95% CI	P	OR_MV_	95% CI	P
Age during therapy	1.005	[1,1.01]	0.0433	1.006	[1,1.01]	0.0624
Sex (male)^a^	0.783	[0.66,0.92]	0.0036	0.824	[0.69,0.98]	0.0273
Ethnicity (UK ethnic minority)^b^	1.216	[0.88,1.68]	0.2368	1.354	[0.97,1.89]	0.0754
University degree	1.755	[1.52,2.02]	0.0001	1.371	[1.18,1.59]	0.0001
Age of onset	1.016	[1.01,1.02]	0.0001	1.001	[0.99,1.01]	0.8761
Number of episodes	0.914	[0.9,0.93]	0.0001	0.945	[0.92,0.97]	0.0001
Number of comorbidities	0.867	[0.82,0.91]	0.0001	0.977	[0.92,1.03]	0.4089
Personality disorder score	0.862	[0.84,0.88]	0.0001	0.887	[0.87,0.91]	0.0001
First therapy	0.866	[0.71,1.05]	0.1524	0.852	[0.69,1.05]	0.1292
Main diagnosis (GAD)^c^	1.155	[1,1.34]	0.0549	1.049	[0.9,1.22]	0.5409
Main diagnosis (PPD)^c^	0.832	[0.66,1.06]	0.1294	1.052	[0.82,1.35]	0.6880
Therapy type (counselling)^d^	1.176	[1.02,1.35]	0.0231	1.110	[0.96,1.28]	0.1575
Therapy type (group)^d^	0.819	[0.62,1.08]	0.1581	0.784	[0.59,1.04]	0.0918
Concurrent medications	0.827	[0.7,0.97]	0.0207	0.985	[0.83,1.17]	0.8640
Regular therapeutic activity	1.441	[1.24,1.68]	0.0001	1.392	[1.19,1.63]	0.0001
Years since therapy at followup	0.977	[0.95,1]	0.0910	0.971	[0.94,1]	0.0376

Reference categories: a, Female; b, white British ethnicity; c, Major Depressive Disorder; d, Cognitive Behavioural Therapy. Note: GAD, generalised anxiety disorder; PPD, phobia or panic disorder

### Sensitivity analyses

Sensitivity analyses showed that there were no statistically significant differences between effect sizes estimated in the primary analyses, which used missing data indicators to retain all participants for analysis (n = 2890), and analyses restricted to participants with complete data (n = 2783; S.Table 13).

When analyses were stratified by timing of therapy relative to GSU-Q, only one notable difference was observed. For participants who received their course of therapy after completing the GSU-Q (n = 1437), concurrent medication use was significantly associated with poorer outcomes, whereas for individuals who received therapy prior to (n = 944), or concurrent to the GSU-Q (n = 701), non-significant effects towards more favourable outcomes were observed (S.Tables 7 and 14).

Finally, we also compared analyses based on different intervals of time (5 years, n = 2511; 10 years, n = 2890; 15 years, n = 3082) between receiving therapy and response to the THO-Q. We observed no statistical differences between effect sizes estimated using these different cut-offs (S.Table 15). This suggests that reliability of recall or changes in therapy protocols has not had a large impact on responses over these time frames.

## Discussion

We tested associations between self-reported therapy outcome and patient characteristics, replicating four associations with therapy outcomes identified using traditional measures of symptom change. Personality disorder symptoms [19, 20]. and the number of recurrent episodes [11] were associated with poorer self-reported outcomes. Higher educational attainment (i.e. obtaining a university degree) and reported regular use of a therapeutic activity were associated with more favourable outcomes.

These findings are in keeping with the previous literature which suggests that more complex cases may require multimodal or long-term support [13]. In previous studies, these markers of case complexity have included personality disorder symptoms, comorbidity and chronicity, of which we found strong evidence only for personality disorder symptoms. Notably, high burden from personality disorder symptoms has been reported in primary care samples in the UK [42]. Whilst there was an association between psychiatric comorbidity and poor treatment outcomes in the univariable model, this association did not remain significant in the multivariable model.

The positive association detected between educational attainment and favourable treatment outcomes is also consistent with prior studies which have found lower cognitive ability to be associated with poorer outcomes following psychological therapy [12]. Reporting use of an additional therapeutic activity was also associated with better outcomes. It makes sense that individuals who used an additional therapeutic activity as well as psychological therapy would experience a greater improvement in symptoms, especially as some of these are evidence-based interventions for depression or anxiety [43]. Additionally, some may have been related to interventions discussed in therapy or part of a relapse-prevention plan agreed at the end of the course of therapy. For example, exercise is an efficacious treatment for mild-to-moderate depression [44] and mindfulness-based cognitive therapy is recommended in the NICE guidelines as an intervention for recurrent depression [45].

Overall, the findings from this study are as expected. All factors tested have small effect sizes and have the expected direction of effect based on the prior literature. While far from conclusive, this pattern of findings suggests that retrospective measures of therapy outcomes may have some value as a supplementary approach towards increasing sample sizes and identifying new predictive factors. Larger samples may provide additional statistical power such that analyses can be stratified into more homogeneous subgroups and allow for interactions between predictive factors to be assessed [23].

Moving forward, it will be particularly important to formally assess the validity of retrospective self-report approaches for measuring therapy outcomes, by collecting concurrent data on symptom severity. The GRC has high test retest reliability [29] and high face validity [30] in studies of chronic pain. However, qualitative research has identified discrepancies between the GRC and PHQ-9 in patients with MDD [46]. They found that participants attributed discrepancies to (1) differences in accuracy between the measures (citing the GRC as more accurate); (2) impact of recent life events at time of measurement; (3) influence of self-motivation (desire for improvement on PHQ9); and (4) poor recall when reporting the GRC.

We acknowledge that retrospective self-report data collected from a volunteer cohort is not a gold-standard approach and is likely to be noisier than clinician rated data, where greater specificity can be achieved. For example, self-reported data is less reliable than clinically ascertained data [47], and selection bias is a well-known limitation of such cohort studies [48]. However, we would argue that a range of approaches to data collection are needed to provide the huge sample sizes that will be required to detect interactions associated with treatment outcomes, in order to develop predictive tools to deliver stratified mental health care.

Another limitation of the study is the time-varying nature of data collection. Ideally, the ordering and intervals between data collection time-points would be the same for all participants. However, we aimed to use a “minimal” broad-brush approach with the ultimate goal of increasing sample size. Notably, when we stratified analyses by approximate timings, we found no striking differences between strata.

In sum, we used a minimal phenotyping approach, a single questionnaire item collected retrospectively, to assess therapy outcomes in a large volunteer cohort of individuals with anxiety or depression. We tested for associations with sociodemographic, clinical and therapy-related factors and replicated three previously reported associations. When traditional approaches are impractical, this approach provides an alternative to collect therapy outcome data quickly and inexpensively on a large scale. However, more work is required to assess the validity of retrospectively self-reported therapy outcome measures. If such measures are highly correlated with traditional outcomes, they may hold promise towards providing the sample sizes that will likely be required to develop predictive tools for stratified mental health care.

## Electronic supplementary material

Below is the link to the electronic supplementary material.

Supplementary Material 1: Patient characteristics associated with prognosis following psychological therapy for anxiety or depressive disorders - a cohort of GLAD study participants

## Data Availability

All analysis code is available from https://github.com/chrisrayner/4_therapy_outcomes_glad. The data that support the findings of this study are available on request from the corresponding author, TCE. The data are not publicly available due to restrictions outlined in the study protocol and specified to participants during the consent process.

## Abbreviations

CBT: Cognitive Behavioural Therapy
CIDI-SF: Composite International Diagnostic Interview Short Form
GAD: Generalized Anxiety Disorder
GAD-7: Generalized Anxiety Disorder 7-item scale
GLAD: Genetic Links to Anxiety and Depression
GSU-Q: GLAD Sign-Up Questionnaire
GRC: Global Rating of Change
MDD: Major Depressive Disorder
PD: Panic Disorder
PHQ-9: Patient Health Questionnaire 9-item version
PPD: Phobias and Panic Disorder
NHS: National Health Service
SoP: Social Phobia
SpP: Specific Phobia
THO-Q: Therapy History and Outcomes Questionnaire
VIF: Variance Inflation Factor

## References

[1] James SL (2018). Global, regional, and national incidence, prevalence, and years lived with disability for 354 diseases and injuries for 195 countries and territories, 1990–2017: a systematic analysis for the Global Burden of Disease Study 2017. Lancet.

[2] Cipriani A (2018). Comparative efficacy and acceptability of 21 antidepressant drugs for the acute treatment of adults with major depressive disorder: a systematic review and network meta-analysis. Lancet.

[3] Cuijpers P, Karyotaki E, de Wit L, Ebert DD (2020). The effects of fifteen evidence-supported therapies for adult depression: A meta-analytic review. Psychother Res.

[4] Community and Mental Health team, NHS Digital. Psychological Therapies Annual report on the use of IAPT services, England 2017-18. *NHS Digital*. (2018).

[5] Slee A (2019). Pharmacological treatments for generalised anxiety disorder: a systematic review and network meta-analysis. Lancet.

[6] El Alaoui S (2016). Predicting Outcome in Internet-Based Cognitive Behaviour Therapy for Major Depression: A Large Cohort Study of Adult Patients in Routine Psychiatric Care. PLoS ONE.

[7] Hollon SD (2014). Effect of cognitive therapy with antidepressant medications vs antidepressants alone on the rate of recovery in major depressive disorder: a randomized clinical trial. JAMA Psychiatry.

[8] Fournier JC (2009). Prediction of response to medication and cognitive therapy in the treatment of moderate to severe depression. J Consult Clin Psychol.

[9] Hölzel L, Härter M, Reese C, Kriston L (2011). Risk factors for chronic depression—a systematic review. J Affect Disord.

[10] Buckman JEJ (2021). The contribution of depressive ‘disorder characteristics’ to determinations of prognosis for adults with depression: an individual patient data meta-analysis. Psychol Med.

[11] Buckman JEJ (2018). Risk factors for relapse and recurrence of depression in adults and how they operate: A four-phase systematic review and meta-synthesis. Clin Psychol Rev.

[12] DeRubeis RJ (2014). The Personalized Advantage Index: Translating Research on Prediction into Individualized Treatment Recommendations. A Demonstration. PLoS ONE.

[13] Delgadillo J, Huey D, Bennett H, McMillan D (2017). Case complexity as a guide for psychological treatment selection. J Consult Clin Psychol.

[14] Saunders R, Cape J, Fearon P, Pilling S (2016). Predicting treatment outcome in psychological treatment services by identifying latent profiles of patients. J Affect Disord.

[15] Buckman JEJ, et al. Indicators of Prognosis Independent of Treatment for Adults with Depression in Primary Care, Going Beyond Baseline Symptom-Severity: A Systematic Review and Individual Patient Data Meta-Analysis. (2020) doi:10.2139/ssrn.3520082.

[16] Dennehy EB, Marangell LB, Martinez J, Balasubramani GK, Wisniewski SR (2014). Clinical and functional outcomes of patients who experience partial response to citalopram: secondary analysis of STAR* D. J Psychiatr Pract.

[17] Angstman KB (2017). Personality Disorders in Primary Care: Impact on Depression Outcomes Within Collaborative Care. J Prim Care Community Health.

[18] Newton-Howes G, Tyrer P, Johnson T (2006). Personality disorder and the outcome of depression: meta-analysis of published studies. Br J Psychiatry.

[19] Newton-Howes G (2014). Influence of personality on the outcome of treatment in depression: systematic review and meta-analysis. J Pers Disord.

[20] Goddard E, Wingrove J, Moran P (2015). The impact of comorbid personality difficulties on response to IAPT treatment for depression and anxiety. Behav Res Ther.

[21] Renaud J, Russell JJ, Myhr G. Predicting who benefits most from cognitive-behavioral therapy for anxiety and depression. J. Clin. Psychol. (2014). 10.1002/jclp.22099 24752934

[22] Simon GE, Perlis RH (2010). Personalized medicine for depression: can we match patients with treatments?. Am J Psychiatry.

[23] Kessler RC (2017). Using patient self-reports to study heterogeneity of treatment effects in major depressive disorder. Epidemiol Psychiatr Sci.

[24] Davies MR (2019). The Genetic Links to Anxiety and Depression (GLAD) Study: Online recruitment into the largest recontactable study of depression and anxiety. Behav Res Ther.

[25] Adamska L (2015). Challenges of linking to routine healthcare records in UK Biobank. Trials.

[26] Cai N (2020). Minimal phenotyping yields genome-wide association signals of low specificity for major depression. Nat Genet.

[27] Sanchez-Roige S, Palmer AA (2020). Emerging phenotyping strategies will advance our understanding of psychiatric genetics. Nat Neurosci.

[28] Howard DM (2018). Genome-wide association study of depression phenotypes in UK Biobank identifies variants in excitatory synaptic pathways. Nat Commun.

[29] Costa LOP (2008). Clinimetric testing of three self-report outcome measures for low back pain patients in Brazil: which one is the best?. Spine.

[30] Fischer D (1999). Capturing the patient’s view of change as a clinical outcome measure. JAMA.

[31] Button KS (2015). Minimal clinically important difference on the Beck Depression Inventory–II according to the patient’s perspective. Psychol Med.

[32] Kroenke K, Spitzer RL, Williams JB (2001). The PHQ-9: validity of a brief depression severity measure. J Gen Intern Med.

[33] Kounali D, et al. How much change is enough? Evidence from a longitudinal study on depression in UK primary care. Psychol. Med. 1–8 (2020). 10.1017/S0033291720003700 PMC9340848 33138872

[34] Kessler RC, Andrews G, Mroczek D, Ustun B, Wittchen H-U (1998). The World Health Organization Composite International Diagnostic Interview short-form (CIDI-SF). Int J Methods Psychiatr Res.

[35] Davis KAS (2019). Mental Health in UK Biobank Revised. Epidemiology.

[36] Olajide K (2018). Development and Psychometric Properties of the Standardized Assessment of Severity of Personality Disorder (SASPD). J Pers Disord.

[37] Harrell F. Regression modelling strategies. https://hbiostat.org/R/rms/ (2020).

[38] Team RC R: A language and environment for statistical computing. R Foundation for Statistical Computing, Vienna, Austria. 2016. (2017).

[39] Brant R (1990). Assessing proportionality in the proportional odds model for ordinal logistic regression. Biometrics.

[40] Nyholt DR (2004). A simple correction for multiple testing for single-nucleotide polymorphisms in linkage disequilibrium with each other. Am J Hum Genet.

[41] Cheverud JM (2001). A simple correction for multiple comparisons in interval mapping genome scans. Heredity.

[42] Hepgul N, et al. Clinical characteristics of patients assessed within an Improving Access to Psychological Therapies (IAPT) service: results from a naturalistic cohort study (Predicting Outcome Following Psychological Therapy; PROMPT). BMC Psychiatry 16, (2016). 10.1186/s12888-016-0736-6 PMC4769576 26920578

[43] Morres ID (2019). Aerobic exercise for adult patients with major depressive disorder in mental health services: A systematic review and meta-analysis. Depress Anxiety.

[44] Choi KW, et al. An Exposure-Wide and Mendelian Randomization Approach to Identifying Modifiable Factors for the Prevention of Depression. Am. J. Psychiatry appiajp202019111158 (2020). 10.1176/appi.ajp.2020.19111158 PMC9361193 32791893

[45] National Collaborating Centre for Mental Health (UK). Depression: The Treatment and Management of Depression in Adults (Updated Edition). British Psychological Society; 2011. 22132433

[46] Robinson J (2017). Why are there discrepancies between depressed patients’ Global Rating of Change and scores on the Patient Health Questionnaire depression module? A qualitative study of primary care in England. BMJ Open.

[47] Moffitt TE (2010). How common are common mental disorders? Evidence that lifetime prevalence rates are doubled by prospective versus retrospective ascertainment. Psychol Med.

[48] Munafò MR, Tilling K, Taylor AE, Evans DM (2018). Davey Smith, G. Collider scope: when selection bias can substantially influence observed associations. Int J Epidemiol.

