# Supplementary material for: Patient characteristics associated with retrospectively self-reported treatment outcomes following psychological therapy for anxiety or depressive disorders - a cohort of GLAD study participants
Source: BMC Psychiatry. 2022 Nov 18;22:719. doi: 10.1186/s12888-022-04275-6 (PMC9675224; doi:10.1186/s12888-022-04275-6)
Supplement: Supplementary file 1 — Supplementary Material 1: Patient characteristics associated with prognosis following psychological therapy for anxiety or depressive disorders - a cohort of GLAD study participants [file 12888_2022_4275_MOESM1_ESM.docx]

Patient characteristics associated with prognosis following psychological therapy for anxiety or depressive disorders - a cohort of GLAD study participants

Rayner et al. 2022

Table of Contents

[**Supplementary tables**](#_heading=h.3rdcrjn) **3**

[Outcomes](#_heading=h.lnxbz9) 3

[Stratified by complete-cases](#_heading=h.1ksv4uv) 3

[Stratified by inclusion criteria](#_heading=h.qkitzaptc1tf) 4

[Analysis variables](#_heading=h.3j2qqm3) 5

[Stratified by complete-cases](#_heading=h.4i7ojhp) 5

[Stratified by inclusion criteria](#_heading=h.3pi4lr1hi0zd) 8

[Additional therapy, anxiety and depression outcomes](#_heading=h.2bn6wsx) 12

[Second therapy outcome](#_heading=h.3as4poj) 12

[Baseline anxiety/depression symptoms](#_heading=h.49x2ik5) 13

[Correlations](#_heading=h.147n2zr) 14

[Sensitivity analyses](#_heading=h.23ckvvd) 16

[Time: therapy relative to sign-up](#_heading=h.32hioqz) 16

[Time: since therapy at time of follow-up](#_heading=h.pug24bq9dics) 18

[Brant test](#_heading=h.vx1227) 19

[VIFS](#_heading=h.1v1yuxt) 20

[Z-tests](#_heading=h.2u6wntf) 21

[**Supplementary figures**](#_heading=h.i2u8ndcwdqgq) **23**

[Participants](#_heading=h.nmf14n) 23

[Time-points](#_heading=h.hpkr2hr53k5l) 24

[Missing data](#_heading=h.n0dw3h516mzy) 26

[Sensitivity analysis](#_heading=h.ysnpnxna459d) 27

[Time: therapy relative to sign-up](#_heading=h.5lafo7va66mb) 27

[Time: since therapy at time of follow-up](#_heading=h.7fx2u8hkg3wr) 28

[**Questionnaire**](#_heading=h.1ekj68ttdoy2) **29**

[Therapy history and outcomes questionnaire"](#_heading=h.imk64ptrb3hu) 29

[Correlations](#_heading=h.k6uvx9f74mqq) 29

# Supplementary tables

## Outcomes

### Stratified by complete-cases

**Title:** S.Table 1. Outcomes (global rating of improvement) following the most recent course of therapy self-reported in a subsample of the Genetic Links to Anxiety and Depression (GLAD) study participants (n=2890) who received psychological therapy (cognitive behavioural therapy or counseling) for major depressive disorder, generalised anxiety disorder, or phobic/panic disorders

Participants are stratified into complete-cases and those with missing data on one or more of the analysis variables. Group differences were compared using chi-square tests

|  | Complete data (N=2783) | Missing analysis data (N=107) | Total (N=2890) | p value |
| --- | --- | --- | --- | --- |
| **Self reported change** |  |  |  | 0.697^1^ |
| Much worse | 67 (2.4%) | 3 (2.8%) | 70 (2.4%) |  |
| A little worse | 109 (3.9%) | 4 (3.7%) | 113 (3.9%) |  |
| No change | 597 (21.5%) | 29 (27.1%) | 626 (21.7%) |  |
| A little better | 1176 (42.3%) | 40 (37.4%) | 1216 (42.1%) |  |
| Much better | 834 (30.0%) | 31 (29.0%) | 865 (29.9%) |  |

###

###

### Stratified by inclusion criteria

**Title:** S.Table 2. Outcomes (global rating of improvement) following the most recent course of therapy self-reported in a subsample of the Genetic Links to Anxiety and Depression (GLAD) study participants (n=2890) who received psychological therapy (cognitive behavioural therapy or counseling) for major depressive disorder, generalised anxiety disorder, or phobic/panic disorders

Participants are stratified into the included analysis sample and those excluded due to one or more of the analysis variables meeting exclusion criteria. Group differences were compared using chi-square tests

|  | Excluded (N=1490) | Included (N=2890) | Total (N=4380) | p value |
| --- | --- | --- | --- | --- |
| **Self reported change** |  |  |  | < 0.001^1^ |
| Much worse | 59 (4.1%) | 70 (2.4%) | 129 (3.0%) |  |
| A little worse | 74 (5.1%) | 113 (3.9%) | 187 (4.3%) |  |
| No change | 369 (25.4%) | 626 (21.7%) | 995 (22.9%) |  |
| A little better | 556 (38.2%) | 1216 (42.1%) | 1772 (40.8%) |  |
| Much better | 397 (27.3%) | 865 (29.9%) | 1262 (29.0%) |  |
| Missing data | 35 | 0 | 35 |  |

## Analysis variables

### Stratified by complete-cases

**Title:** S.Table 3. Sociodemographic, clinical and therapy factors self-reported in a subsample of the Genetic Links to Anxiety and Depression (GLAD) study participants (n=2890) who received psychological therapy (cognitive behavioural therapy or counseling) for major depressive disorder, generalised anxiety disorder, or phobic/panic disorders

Participants are stratified into complete-cases and those with missing data on one or more of the analysis variables. Group differences were compared using chi-square tests and t-tests

|  | **Complete data (N=2783)** | **Missing analysis data (N=107)** | **Total (N=2890)** | p value |
| --- | --- | --- | --- | --- |
| **Age at signup** |  |  |  | 0.102^1^ |
| Mean (SD) | 39.2 (13.7) | 41.4 (14.3) | 39.3 (13.7) |  |
| Range | 16.4 - 79.9 | 17.0 - 71.9 | 16.4 - 79.9 |  |
| **Age during therapy** |  |  |  | 0.125^1^ |
| Mean (SD) | 38.2 (13.4) | 40.2 (13.9) | 38.3 (13.4) |  |
| Range | 18.0 - 79.0 | 18.0 - 72.0 | 18.0 - 79.0 |  |
| **Sex** |  |  |  | 0.067^2^ |
| Female | 2207 (79.3%) | 77 (72.0%) | 2284 (79.0%) |  |
| Male | 576 (20.7%) | 30 (28.0%) | 606 (21.0%) |  |
| **Ethnicity** |  |  |  | 0.093^2^ |
| Arab | 2 (0.1%) | 0 (0.0%) | 2 (0.1%) |  |
| Asian or asian british | 24 (0.9%) | 3 (2.8%) | 27 (0.9%) |  |
| Black or black british | 9 (0.3%) | 0 (0.0%) | 9 (0.3%) |  |
| Mixed | 69 (2.5%) | 2 (1.9%) | 71 (2.5%) |  |
| Other | 23 (0.8%) | 3 (2.8%) | 26 (0.9%) |  |
| White | 2652 (95.4%) | 98 (92.5%) | 2750 (95.3%) |  |
| Missing data | 4 | 1 | 5 |  |
| **University degree** |  |  |  | 0.648^2^ |
| No | 1003 (36.4%) | 33 (38.8%) | 1036 (36.5%) |  |
| Yes | 1752 (63.6%) | 52 (61.2%) | 1804 (63.5%) |  |
| Missing data | 28 | 22 | 50 |  |
| **Age of onset** |  |  |  | < 0.001^1^ |
| Mean (SD) | 18.8 (9.4) | 24.7 (12.9) | 18.9 (9.5) |  |
| Range | 5.0 - 61.0 | 6.0 - 58.0 | 5.0 - 61.0 |  |
| Missing data | 130 | 65 | 195 |  |
| **Number of episodes** |  |  |  | 0.004^1^ |
| Mean (SD) | 9.1 (3.7) | 11.4 (3.1) | 9.1 (3.7) |  |
| Range | 1.0 - 13.0 | 3.0 - 13.0 | 1.0 - 13.0 |  |
| Missing data | 610 | 85 | 695 |  |
| **Number of comorbidities** |  |  |  | 0.812^1^ |
| Mean (SD) | 1.4 (1.3) | 1.4 (1.5) | 1.4 (1.3) |  |
| Range | 0.0 - 6.0 | 0.0 - 6.0 | 0.0 - 6.0 |  |
| **Personality disorder score** |  |  |  | 0.347^1^ |
| Mean (SD) | 8.1 (3.5) | 7.8 (3.7) | 8.1 (3.5) |  |
| Range | 0.0 - 23.0 | 1.0 - 18.0 | 0.0 - 23.0 |  |
| Missing data | 0 | 1 | 1 |  |
| **First therapy** |  |  |  | 0.143^2^ |
| No | 2417 (87.0%) | 82 (82.0%) | 2499 (86.9%) |  |
| Yes | 360 (13.0%) | 18 (18.0%) | 378 (13.1%) |  |
| Missing data | 6 | 7 | 13 |  |
| **Years since therapy at followup** |  |  |  | 0.964^1^ |
| Mean (SD) | 2.5 (2.5) | 2.5 (2.5) | 2.5 (2.5) |  |
| Range | 0.0 - 10.0 | 0.0 - 10.0 | 0.0 - 10.0 |  |
| **Main diagnosis** |  |  |  |  |
| Major depressive disorder | 1667 (59.9%) | 44 (41.1%) | 1711 (59.2%) |  |
| Generalised anxiety disorder | 873 (31.4%) | 50 (46.7%) | 923 (31.9%) |  |
| Phobia (various) | 41 (1.5%) | 1 (0.9%) | 42 (1.5%) |  |
| Social anxiety disorder | 133 (4.8%) | 9 (8.4%) | 142 (4.9%) |  |
| Panic disorder | 69 (2.5%) | 3 (2.8%) | 72 (2.5%) |  |
| Bipolar disorder | 0 (0.0%) | 0 (0.0%) | 0 (0.0%) |  |
| Don’t know | 0 (0.0%) | 0 (0.0%) | 0 (0.0%) |  |
| Eating disorder | 0 (0.0%) | 0 (0.0%) | 0 (0.0%) |  |
| Other | 0 (0.0%) | 0 (0.0%) | 0 (0.0%) |  |
| Personality disorder | 0 (0.0%) | 0 (0.0%) | 0 (0.0%) |  |
| Post traumatic stress disorder | 0 (0.0%) | 0 (0.0%) | 0 (0.0%) |  |
| Prefer not to answer | 0 (0.0%) | 0 (0.0%) | 0 (0.0%) |  |
| **Therapy type** |  |  |  |  |
| One-to-one cbt | 1159 (41.6%) | 52 (48.6%) | 1211 (41.9%) |  |
| One-to-one counselling | 1432 (51.5%) | 51 (47.7%) | 1483 (51.3%) |  |
| Group cbt | 159 (5.7%) | 3 (2.8%) | 162 (5.6%) |  |
| Group counselling | 33 (1.2%) | 1 (0.9%) | 34 (1.2%) |  |
| Group other | 0 (0.0%) | 0 (0.0%) | 0 (0.0%) |  |
| Individual other | 0 (0.0%) | 0 (0.0%) | 0 (0.0%) |  |
| **Concurrent medications** |  |  |  | < 0.001^2^ |
| No | 626 (22.6%) | 39 (41.1%) | 665 (23.2%) |  |
| Yes | 2150 (77.4%) | 56 (58.9%) | 2206 (76.8%) |  |
| Missing data | 7 | 12 | 19 |  |
| **Regular therapeutic activity** |  |  |  | 0.044^2^ |
| No | 762 (28.1%) | 28 (38.9%) | 790 (28.3%) |  |
| Yes | 1953 (71.9%) | 44 (61.1%) | 1997 (71.7%) |  |
| Missing data | 68 | 35 | 103 |  |
| **Anxiety score at baseline** |  |  |  | 0.926^1^ |
| Mean (SD) | 9.1 (5.9) | 9.1 (5.8) | 9.1 (5.9) |  |
| Range | 0.0 - 21.0 | 0.0 - 21.0 | 0.0 - 21.0 |  |
| **Depression score at baseline** |  |  |  | 0.085^1^ |
| Mean (SD) | 13.7 (8.3) | 12.3 (8.9) | 13.6 (8.3) |  |
| Range | 0.0 - 31.0 | 0.0 - 31.0 | 0.0 - 31.0 |  |
| **In remission at baseline** |  |  |  | 0.165^2^ |
| No | 1845 (66.3%) | 64 (59.8%) | 1909 (66.1%) |  |
| Yes | 938 (33.7%) | 43 (40.2%) | 981 (33.9%) |  |

###

### Stratified by inclusion criteria

**Title:** S.Table 4. Sociodemographic, clinical and therapy factors self-reported in a subsample of the Genetic Links to Anxiety and Depression (GLAD) study participants who received psychological therapy (cognitive behavioural therapy or counseling) for major depressive disorder, generalised anxiety disorder, or phobic/panic disorders

Participants are stratified into the included analysis sample (n=2890) and those excluded due to one or more of the analysis variables meeting exclusion criteria (n=1298) and GLAD participants who did not participate in the follow-up questionnaire (n=33033. Group differences were compared using chi-square tests

|  | **Did not participate (N=33033)** | **Excluded (N=1490)** | **Included (N=2890)** | **Total (N=37413)** | p value |
| --- | --- | --- | --- | --- | --- |
| **Age at signup** |  |  |  |  | < 0.001^1^ |
| Mean (SD) | 37.4 (14.4) | 40.3 (16.2) | 39.3 (13.7) | 37.6 (14.5) |  |
| Range | -0.8 - 118.7 | 15.0 - 92.3 | 16.4 - 79.9 | -0.8 - 118.7 |  |
| Missing data | 44 | 35 | 0 | 79 |  |
| **Age during therapy** |  |  |  |  |  |
| Mean (SD) | NA | 34.2 (13.7) | 38.3 (13.4) | 36.9 (13.6) |  |
| Range | NA | 4.0 - 74.0 | 18.0 - 79.0 | 4.0 - 79.0 |  |
| Missing data | 33033 | 9 | 0 | 33042 |  |
| **Sex** |  |  |  |  | 0.002^2^ |
| Female | 26233 (79.4%) | 1212 (83.2%) | 2284 (79.0%) | 29729 (79.6%) |  |
| Male | 6789 (20.6%) | 244 (16.8%) | 606 (21.0%) | 7639 (20.4%) |  |
| Missing data | 11 | 34 | 0 | 45 |  |
| **Ethnicity** |  |  |  |  | 0.133^2^ |
| Arab | 32 (0.1%) | 0 (0.0%) | 2 (0.1%) | 34 (0.1%) |  |
| Asian or asian british | 468 (1.4%) | 10 (0.7%) | 27 (0.9%) | 505 (1.4%) |  |
| Black or black british | 168 (0.5%) | 8 (0.5%) | 9 (0.3%) | 185 (0.5%) |  |
| Mixed | 840 (2.6%) | 32 (2.2%) | 71 (2.5%) | 943 (2.5%) |  |
| Other | 321 (1.0%) | 14 (1.0%) | 26 (0.9%) | 361 (1.0%) |  |
| White | 30957 (94.4%) | 1391 (95.6%) | 2750 (95.3%) | 35098 (94.5%) |  |
| Missing data | 247 | 35 | 5 | 287 |  |
| **University degree** |  |  |  |  | < 0.001^2^ |
| No | 14862 (46.4%) | 619 (44.2%) | 1036 (36.5%) | 16517 (45.5%) |  |
| Yes | 17184 (53.6%) | 782 (55.8%) | 1804 (63.5%) | 19770 (54.5%) |  |
| Missing data | 987 | 89 | 50 | 1126 |  |
| **Age of onset** |  |  |  |  | < 0.001^1^ |
| Mean (SD) | 18.2 (9.1) | 17.4 (8.6) | 18.9 (9.5) | 18.3 (9.1) |  |
| Range | 5.0 - 79.0 | 5.0 - 66.0 | 5.0 - 61.0 | 5.0 - 79.0 |  |
| Missing data | 3943 | 139 | 195 | 4277 |  |
| **Number of episodes** |  |  |  |  | 0.003^1^ |
| Mean (SD) | 9.1 (3.7) | 9.5 (3.7) | 9.1 (3.7) | 9.2 (3.7) |  |
| Range | 1.0 - 13.0 | 2.0 - 13.0 | 1.0 - 13.0 | 1.0 - 13.0 |  |
| Missing data | 8855 | 328 | 695 | 9878 |  |
| **Number of comorbidities** |  |  |  |  | < 0.001^1^ |
| Mean (SD) | 0.0 (0.0) | 1.5 (1.5) | 1.4 (1.3) | 1.3 (1.4) |  |
| Range | 0.0 - 0.0 | 0.0 - 6.0 | 0.0 - 6.0 | 0.0 - 6.0 |  |
| Missing data | 32730 | 0 | 0 | 32730 |  |
| **Personality disorder score** |  |  |  |  | 0.002^1^ |
| Mean (SD) | 8.4 (3.7) | 8.5 (3.8) | 8.1 (3.5) | 8.3 (3.7) |  |
| Range | 0.0 - 27.0 | 0.0 - 25.0 | 0.0 - 23.0 | 0.0 - 27.0 |  |
| Missing data | 2372 | 34 | 1 | 2407 |  |
| **First therapy** |  |  |  |  |  |
| No | 0 | 1228 (82.9%) | 2499 (86.9%) | 3727 (85.5%) |  |
| Yes | 0 | 254 (17.1%) | 378 (13.1%) | 632 (14.5%) |  |
| Missing data | 33033 | 8 | 13 | 33054 |  |
| **Years since therapy at followup** |  |  |  |  |  |
| Mean (SD) | NA | 7.6 (8.9) | 2.5 (2.5) | 4.2 (6.0) |  |
| Range | NA | 0.0 - 64.0 | 0.0 - 10.0 | 0.0 - 64.0 |  |
| Missing data | 33033 | 44 | 0 | 33077 |  |
| **Main diagnosis** |  |  |  |  |  |
| Major depressive disorder | 0 | 551 (37.3%) | 1711 (59.2%) | 2262 (51.8%) |  |
| Generalised anxiety disorder | 0 | 182 (12.3%) | 923 (31.9%) | 1105 (25.3%) |  |
| Phobia (various) | 0 | 13 (0.9%) | 42 (1.5%) | 55 (1.3%) |  |
| Social anxiety disorder | 0 | 33 (2.2%) | 142 (4.9%) | 175 (4.0%) |  |
| Panic disorder | 0 | 33 (2.2%) | 72 (2.5%) | 105 (2.4%) |  |
| Bipolar disorder | 0 | 40 (2.7%) | 0 (0.0%) | 40 (0.9%) |  |
| Don’t know | 0 | 81 (5.5%) | 0 (0.0%) | 81 (1.9%) |  |
| Eating disorder | 0 | 71 (4.8%) | 0 (0.0%) | 71 (1.6%) |  |
| Other | 0 | 192 (13.0%) | 0 (0.0%) | 192 (4.4%) |  |
| Personality disorder | 0 | 134 (9.1%) | 0 (0.0%) | 134 (3.1%) |  |
| Post traumatic stress disorder | 0 | 135 (9.1%) | 0 (0.0%) | 135 (3.1%) |  |
| Prefer not to answer | 0 | 11 (0.7%) | 0 (0.0%) | 11 (0.3%) |  |
| Missing data | 33033 | 14 | 0 | 33047 |  |
| **Therapy type** |  |  |  |  |  |
| One-to-one cbt | 0 | 400 (28.1%) | 1211 (41.9%) | 1611 (37.4%) |  |
| One-to-one counselling | 0 | 568 (40.0%) | 1483 (51.3%) | 2051 (47.6%) |  |
| Group cbt | 0 | 73 (5.1%) | 162 (5.6%) | 235 (5.5%) |  |
| Group counselling | 0 | 37 (2.6%) | 34 (1.2%) | 71 (1.6%) |  |
| Group other | 0 | 86 (6.1%) | 0 (0.0%) | 86 (2.0%) |  |
| Individual other | 0 | 257 (18.1%) | 0 (0.0%) | 257 (6.0%) |  |
| Missing data | 33033 | 69 | 0 | 33102 |  |
| **Concurrent medications** |  |  |  |  |  |
| No | 0 | 430 (29.9%) | 665 (23.2%) | 1095 (25.4%) |  |
| Yes | 0 | 1007 (70.1%) | 2206 (76.8%) | 3213 (74.6%) |  |
| Missing data | 33033 | 53 | 19 | 33105 |  |
| **Regular therapeutic activity** |  |  |  |  | < 0.001^2^ |
| No | 179 (60.9%) | 434 (30.5%) | 790 (28.3%) | 1403 (31.1%) |  |
| Yes | 115 (39.1%) | 990 (69.5%) | 1997 (71.7%) | 3102 (68.9%) |  |
| Missing data | 32739 | 66 | 103 | 32908 |  |
| **Anxiety score at baseline** |  |  |  |  | 0.771^1^ |
| Mean (SD) | 9.1 (6.3) | 9.3 (6.2) | 9.1 (5.9) | 9.1 (6.2) |  |
| Range | 0.0 - 21.0 | 0.0 - 21.0 | 0.0 - 21.0 | 0.0 - 21.0 |  |
| Missing data | 11 | 34 | 0 | 45 |  |
| **Depression score at baseline** |  |  |  |  | 0.247^1^ |
| Mean (SD) | 13.6 (8.3) | 14.0 (8.5) | 13.6 (8.3) | 13.6 (8.4) |  |
| Range | 0.0 - 31.0 | 0.0 - 31.0 | 0.0 - 31.0 | 0.0 - 31.0 |  |
| Missing data | 11 | 34 | 0 | 45 |  |
| **In remission at baseline** |  |  |  |  | 0.150^2^ |
| No | 22396 (67.8%) | 987 (67.8%) | 1909 (66.1%) | 25292 (67.7%) |  |
| Yes | 10626 (32.2%) | 469 (32.2%) | 981 (33.9%) | 12076 (32.3%) |  |
| Missing data | 11 | 34 | 0 | 45 |  |

## Additional therapy, anxiety and depression outcomes

### Second therapy outcome

**Title:** S.Table 5. Cross-tabulation of outcomes (global rating of improvement) following the **first** and **second** most recent courses of therapy self-reported in a subsample of the Genetic Links to Anxiety and Depression (GLAD) study participants who received psychological therapy (cognitive behavioural therapy or counseling) for major depressive disorder, generalised anxiety disorder, or phobic/panic disorders **more than once**

|  | **Much worse (N=108)** | **A little worse (N=146)** | **No change (N=680)** | **A little better (N=955)** | **Much better (N=573)** | **Total (N=2462)** |
| --- | --- | --- | --- | --- | --- | --- |
| **Self reported change** |  |  |  |  |  |  |
| Much worse | 18 (16.7%) | 9 (6.2%) | 15 (2.2%) | 9 (0.9%) | 8 (1.4%) | 59 (2.4%) |
| A little worse | 19 (17.6%) | 15 (10.3%) | 35 (5.1%) | 18 (1.9%) | 9 (1.6%) | 96 (3.9%) |
| No change | 23 (21.3%) | 46 (31.5%) | 253 (37.2%) | 146 (15.3%) | 52 (9.1%) | 520 (21.1%) |
| A little better | 28 (25.9%) | 46 (31.5%) | 249 (36.6%) | 492 (51.5%) | 222 (38.7%) | 1037 (42.1%) |
| Much better | 20 (18.5%) | 30 (20.5%) | 128 (18.8%) | 290 (30.4%) | 282 (49.2%) | 750 (30.5%) |
| **In remission at baseline** |  |  |  |  |  |  |
| No | 90 (83.3%) | 120 (82.2%) | 525 (77.2%) | 611 (64.0%) | 304 (53.1%) | 1650 (67.0%) |
| Yes | 18 (16.7%) | 26 (17.8%) | 155 (22.8%) | 344 (36.0%) | 269 (46.9%) | 812 (33.0%) |

### Baseline anxiety/depression symptoms

**Title:** S.Table 6. Cross-tabulation of outcomes following the most recent course of therapy (global rating of improvement) recorded at follow-up and measures of anxiety (GAD-7) and depression (PHQ-9) recorded at baseline sign-up, self-reported in a subsample of the Genetic Links to Anxiety and Depression (GLAD) study participants who received psychological therapy (cognitive behavioural therapy or counseling) for major depressive disorder, generalised anxiety disorder, or phobic/panic disorders **more than once**

*Note*: “In remission at baseline (yes)” describes participants that were below the diagnostic threshold for **both** generalised anxiety disorder (GAD-7) and major depressive disorder (PHQ-9) at **baseline recruitement** into the GLAD study

|  | Much worse (N=70) | A little worse (N=113) | No change (N=626) | A little better (N=1216) | Much better (N=865) | Total (N=2890) |
| --- | --- | --- | --- | --- | --- | --- |
| **Anxiety score at baseline** |  |  |  |  |  |  |
| Mean (SD) | 11.4 (6.2) | 11.7 (6.3) | 10.6 (6.1) | 9.1 (5.7) | 7.5 (5.4) | 9.1 (5.9) |
| Median | 11.0 | 11.0 | 10.0 | 8.0 | 6.0 | 8.0 |
| Range | 1.0 - 21.0 | 0.0 - 21.0 | 0.0 - 21.0 | 0.0 - 21.0 | 0.0 - 21.0 | 0.0 - 21.0 |
| **Depression score at baseline** |  |  |  |  |  |  |
| Mean (SD) | 18.9 (9.7) | 18.7 (8.5) | 16.6 (8.1) | 13.5 (8.0) | 10.5 (7.5) | 13.6 (8.3) |
| Median | 19.5 | 19.0 | 16.0 | 13.0 | 9.0 | 13.0 |
| Range | 0.0 - 31.0 | 0.0 - 31.0 | 0.0 - 31.0 | 0.0 - 31.0 | 0.0 - 31.0 | 0.0 - 31.0 |
| **In remission at baseline** |  |  |  |  |  |  |
| No | 57 (81.4%) | 94 (83.2%) | 499 (79.7%) | 810 (66.6%) | 449 (51.9%) | 1909 (66.1%) |
| Yes | 13 (18.6%) | 19 (16.8%) | 127 (20.3%) | 406 (33.4%) | 416 (48.1%) | 981 (33.9%) |

## Correlations

**Title:** S.Table 9. Correlations between sociodemographic, clinical and therapy factors self-reported in a subsample of the Genetic Links to Anxiety and Depression (GLAD) study participants (n=2890) who received psychological therapy (cognitive behavioural therapy or counseling) for major depressive disorder, generalised anxiety disorder, or phobic/panic disorders

*Correlations were used to calculate the number of effectively independent tests, by computing the number of principal components explaining 95% of the variance from the correlation matrix.*

| **Variable 1** | **Variable 2** | **r** | **se** |
| --- | --- | --- | --- |
| In remission at baseline | Depression score at baseline | -0.96 | 0.001 |
| Age during therapy | Age at signup | 0.91 | 0.003 |
| In remission at baseline | Anxiety score at baseline | -0.84 | 0.003 |
| Depression score at baseline | Anxiety score at baseline | 0.68 | 0.003 |
| In remission at baseline | Personality disorder score | -0.60 | 0.005 |
| Depression score at baseline | Personality disorder score | 0.55 | 0.004 |
| Anxiety score at baseline | Personality disorder score | 0.54 | 0.004 |
| Age of onset | Age at signup | 0.42 | 0.005 |
| Age of onset | Age during therapy | 0.40 | 0.013 |
| Years since therapy at followup | Age at signup | 0.36 | 0.013 |
| Depression score at baseline | Number of episodes | 0.32 | 0.005 |
| In remission at baseline | Number of episodes | -0.31 | 0.007 |
| Regular therapeutic activity | First therapy | -0.31 | 0.028 |
| Personality disorder score | Number of episodes | 0.30 | 0.006 |
| In remission at baseline | Number of comorbidities | -0.30 | 0.018 |
| Anxiety score at baseline | Number of comorbidities | 0.29 | 0.013 |
| Personality disorder score | Number of comorbidities | 0.28 | 0.013 |
| Depression score at baseline | Number of comorbidities | 0.27 | 0.014 |
| Personality disorder score | Age of onset | -0.26 | 0.005 |
| In remission at baseline | Age of onset | 0.26 | 0.006 |
| Personality disorder score | University degree | -0.25 | 0.006 |
| Number of episodes | Age of onset | -0.25 | 0.006 |
| In remission at baseline | Age at signup | 0.24 | 0.006 |
| Sex | Age during therapy | 0.24 | 0.020 |
| Depression score at baseline | Age of onset | -0.24 | 0.005 |
| Anxiety score at baseline | Number of episodes | 0.24 | 0.006 |
| Depression score at baseline | University degree | -0.23 | 0.006 |
| In remission at baseline | University degree | 0.23 | 0.008 |
| Years since therapy at followup | First therapy | 0.22 | 0.019 |
| Anxiety score at baseline | Age of onset | -0.21 | 0.005 |
| Sex | Age at signup | 0.20 | 0.007 |
| Anxiety score at baseline | Age at signup | -0.20 | 0.005 |
| Ethnicity | Age during therapy | 0.20 | 0.034 |
| Concurrent medications | Number of comorbidities | 0.20 | 0.020 |
| Ethnicity | Age at signup | 0.19 | 0.011 |
| Personality disorder score | Age at signup | -0.19 | 0.005 |
| Depression score at baseline | Age at signup | -0.19 | 0.005 |
| In remission at baseline | Age during therapy | 0.19 | 0.019 |
| Age of onset | Sex | 0.19 | 0.007 |
| Depression score at baseline | Concurrent medications | 0.18 | 0.020 |
| Anxiety score at baseline | Age during therapy | -0.17 | 0.015 |
| Regular therapeutic activity | Sex | -0.17 | 0.027 |
| Anxiety score at baseline | University degree | -0.16 | 0.006 |
| Concurrent medications | First therapy | -0.16 | 0.030 |
| Regular therapeutic activity | Years since therapy at followup | -0.16 | 0.019 |
| In remission at baseline | Years since therapy at followup | 0.16 | 0.019 |
| Concurrent medications | Main diagnosis | -0.16 | 0.022 |

## Sensitivity analyses

### Time: therapy relative to sign-up

**Title:** S.Table 7. Summary statistics from multivariable (MV) proportional odds ordinal logistic regression models using maximum likelihood estimation to test for associations between self-rated therapy outcomes (global rating of improvement) and sociodemographic, clinical and therapy factors self-reported in a subsample of the Genetic Links to Anxiety and Depression (GLAD) study participants (n=2890) who received psychological therapy (cognitive behavioural therapy or counseling) for major depressive disorder, generalised anxiety disorder, or phobic/panic disorders. *Analyses were stratified by approximate timing of therapy relative to timing of the baseline sign up questionnaire.* Odds ratios, 95% confidence intervals and p-values; *Order from left to right:* ***1. Full unstratified model (main analysis reported in manuscript - provided for easy comparison; n=2890); 2.“Prior” stratum - i.e. those who received their most recent course of therapy prior to signing up and completing the baseline questionnaire; n=944); 3. “Concurrent” stratum - i.e. those who were receiving their most recent course of therapy approximately concurrently to signing up and completing the baseline questionnaire; n=701); 4. “Post” stratum - i.e. those who received their most recent course of therapy after signing up and completing the baseline questionnaire; n=1437).*** *See S.Figure 2 for more details*

| **Inclusion criteria:** | **Full [main analysis]** (n=2890) | | | **Therapy prior to sign-up**  (n=944) | | | **Therapy concurrent to sign-up** (n=701) | | | **Therapy after sign-up** (n=1437) | | |
| --- | --- | --- | --- | --- | --- | --- | --- | --- | --- | --- | --- | --- |
| *Variable* | OR_FULL_ | 95% CI | P | OR_PRIOR_ | 95% CI | P | OR_CONC_ | 95% CI | P | OR_POST_ | 95% CI | P |
| Age during therapy | 1.006 | [1,1.01] | 0.0583 | 1.004 | [0.99,1.01] | 0.4268 | 1.003 | [0.99,1.01] | 0.6242 | 1.008 | [1,1.02] | 0.0625 |
| Sex (male) | 0.822 | [0.69,0.98] | 0.0259 | 0.749 | [0.55,1.02] | 0.0669 | 0.760 | [0.53,1.09] | 0.1400 | 0.896 | [0.7,1.14] | 0.3667 |
| UK ethnic minority | 1.347 | [0.96,1.88] | 0.0798 | 1.191 | [0.67,2.11] | 0.5491 | 1.939 | [0.97,3.88] | 0.0613 | 1.091 | [0.66,1.82] | 0.7376 |
| University degree | 1.368 | [1.18,1.59] | 0.0001 | 1.393 | [1.07,1.81] | 0.0126 | 1.651 | [1.22,2.23] | 0.0012 | 1.230 | [1,1.52] | 0.0557 |
| Age of onset | 1.001 | [0.99,1.01] | 0.8812 | 1.009 | [0.99,1.03] | 0.3261 | 1.000 | [0.98,1.02] | 0.9696 | 0.996 | [0.98,1.01] | 0.5272 |
| Number of episodes | 0.945 | [0.92,0.97] | 0.0001 | 0.951 | [0.91,0.99] | 0.0196 | 0.927 | [0.89,0.97] | 0.0013 | 0.952 | [0.92,0.98] | 0.0024 |
| Number of comorbidities | 0.978 | [0.92,1.03] | 0.4382 | 1.012 | [0.92,1.12] | 0.8186 | 0.902 | [0.81,1.01] | 0.0672 | 0.991 | [0.91,1.08] | 0.8235 |
| Personality disorder score | 0.887 | [0.87,0.91] | 0.0001 | 0.861 | [0.83,0.9] | 0.0001 | 0.898 | [0.86,0.94] | 0.0001 | 0.895 | [0.87,0.92] | 0.0001 |
| First therapy | 0.852 | [0.69,1.05] | 0.1296 | 0.817 | [0.51,1.3] | 0.3959 | 0.767 | [0.51,1.16] | 0.2088 | 0.878 | [0.68,1.14] | 0.3232 |
| Therapy type (counselling) | 1.111 | [0.96,1.28] | 0.1522 | 1.240 | [0.96,1.61] | 0.1066 | 1.121 | [0.83,1.51] | 0.4522 | 0.954 | [0.78,1.17] | 0.6516 |
| Therapy type (group) | 0.784 | [0.59,1.04] | 0.0920 | 0.700 | [0.4,1.22] | 0.2064 | 0.541 | [0.3,0.96] | 0.0355 | 1.002 | [0.69,1.46] | 0.9928 |
| Concurrent medications | 0.982 | [0.83,1.16] | 0.8380 | 1.216 | [0.9,1.64] | 0.2018 | 1.232 | [0.87,1.74] | 0.2378 | 0.693 | [0.54,0.88] | 0.0032 |
| Regular therapeutic activity | 1.394 | [1.19,1.63] | 0.0001 | 1.378 | [1.02,1.86] | 0.0347 | 1.406 | [1.03,1.92] | 0.0333 | 1.368 | [1.1,1.7] | 0.0041 |
| Main diagnosis (gad) | 1.050 | [0.9,1.22] | 0.5320 | 1.337 | [1.02,1.75] | 0.0344 | 1.059 | [0.77,1.45] | 0.7212 | 0.860 | [0.69,1.08] | 0.1893 |
| Main diagnosis (ppd) | 1.050 | [0.82,1.34] | 0.7016 | 1.110 | [0.7,1.77] | 0.6599 | 1.148 | [0.7,1.89] | 0.5898 | 0.899 | [0.64,1.27] | 0.5446 |
| Years since therapy | 0.971 | [0.94,1] | 0.0368 | 1.034 | [0.81,1.32] | 0.7912 | 0.811 | [0.66,1] | 0.0553 | 0.965 | [0.94,0.99] | 0.0107 |

### Time: since therapy at time of follow-up

**Title:** S.Table 8. Summary statistics from multivariable (MV) proportional odds ordinal logistic regression models using maximum likelihood estimation to test for associations between self-rated therapy outcomes (global rating of improvement) and sociodemographic, clinical and therapy factors self-reported in a subsample of the Genetic Links to Anxiety and Depression (GLAD) study participants (n=2890) who received psychological therapy (cognitive behavioural therapy or counseling) for major depressive disorder, generalised anxiety disorder, or phobic/panic disorders. *Threshold for inclusion in the main analysis was 10 years time difference between when the participant received therapy and when they completed the follow-up questionnaire (2010-2020). Sensitivity analyses were performed using two additional thresholds: 5 years (2015-2020) and 15 years (2005-2020).* Odds ratios, 95% confidence intervals and p-values; Order from left to right: **1. 5 years (n=2511); 2. 10 years (main analysis reported in manuscript - provided for easy comparison; n=2890); 3. 15 years (n=3082).**

| **Inclusion criteria:** | **5 year cut-off (n=2511)** | | | **10 year cut-off (n=2890)** | | | **15 year cut-off (n=3082)** | | |
| --- | --- | --- | --- | --- | --- | --- | --- | --- | --- |
| Variable | OR_5y_ | 95% CI | P | OR_10y_ | 95% CI | P | OR_15y_ | 95% CI | P |
| Age during therapy | 1.006 | [1,1.01] | 0.0617 | 1.006 | [1,1.01] | 0.0583 | 1.006 | [1,1.01] | 0.0479 |
| Sex (male) | 0.761 | [0.63,0.92] | 0.0042 | 0.822 | [0.69,0.98] | 0.0259 | 0.825 | [0.7,0.97] | 0.0227 |
| Ethnicity (uk ethnic minority) | 1.379 | [0.96,1.98] | 0.0795 | 1.347 | [0.96,1.88] | 0.0798 | 1.317 | [0.95,1.83] | 0.1005 |
| University degree | 1.342 | [1.15,1.57] | 0.0003 | 1.368 | [1.18,1.59] | 0.0001 | 1.358 | [1.18,1.57] | 0.0001 |
| Age of onset | 1.002 | [0.99,1.01] | 0.7049 | 1.001 | [0.99,1.01] | 0.8812 | 1.000 | [0.99,1.01] | 0.9407 |
| Number of episodes | 0.948 | [0.92,0.97] | 0.0001 | 0.945 | [0.92,0.97] | 0.0001 | 0.946 | [0.92,0.97] | 0.0001 |
| Number of comorbidities | 0.951 | [0.9,1.01] | 0.0999 | 0.978 | [0.92,1.03] | 0.4382 | 0.974 | [0.92,1.03] | 0.3389 |
| Personality disorder score | 0.883 | [0.86,0.9] | 0.0001 | 0.887 | [0.87,0.91] | 0.0001 | 0.888 | [0.87,0.91] | 0.0001 |
| First therapy | 0.801 | [0.64,1.01] | 0.0601 | 0.852 | [0.69,1.05] | 0.1296 | 0.837 | [0.69,1.02] | 0.0761 |
| Therapy type (counselling) | 1.130 | [0.97,1.32] | 0.1229 | 1.111 | [0.96,1.28] | 0.1522 | 1.076 | [0.94,1.24] | 0.3004 |
| Therapy type (group) | 0.683 | [0.5,0.93] | 0.0150 | 0.784 | [0.59,1.04] | 0.0920 | 0.765 | [0.58,1] | 0.0528 |
| Concurrent medications | 1.076 | [0.9,1.29] | 0.4291 | 0.982 | [0.83,1.16] | 0.8380 | 0.943 | [0.8,1.11] | 0.4855 |
| Regular therapeutic activity | 1.300 | [1.1,1.54] | 0.0025 | 1.394 | [1.19,1.63] | 0.0001 | 1.372 | [1.18,1.6] | 0.0001 |
| Main diagnosis (gad) | 1.089 | [0.92,1.28] | 0.3065 | 1.050 | [0.9,1.22] | 0.5320 | 1.033 | [0.89,1.2] | 0.6708 |
| Main diagnosis (ppd) | 1.094 | [0.83,1.43] | 0.5168 | 1.050 | [0.82,1.34] | 0.7016 | 1.009 | [0.79,1.28] | 0.9385 |
| Years since therapy at followup | 0.948 | [0.9,1] | 0.0479 | 0.971 | [0.94,1] | 0.0368 | 0.968 | [0.95,0.99] | 0.0012 |

## Brant test

**Title:** S.Table 10. Summary statistics (chi-square and p-values) from a Brant test to assess the assumption of outcome proportionality for all explanatory variables included in the proportional odds model. The null hypothesis is that the parallel regression assumption holds - i.e. p<0.05 indicates a violation of this assumption

| **Variable** | **X2** | **df** | **probability** |
| --- | --- | --- | --- |
| Concurrent medications | 7.7497597 | 3 | 0.0514767 |
| Therapy type: Counselling | 7.2834178 | 3 | 0.0633925 |
| Main diagnosis: PPD | 5.7973697 | 3 | 0.1218957 |
| First therapy | 5.0412576 | 3 | 0.1688009 |
| Regular therapeutic activity | 3.9898792 | 3 | 0.2625591 |
| Age of onset | 3.9302888 | 3 | 0.2690907 |
| Age during therapy | 3.7872785 | 3 | 0.2853693 |
| Omnibus | 49.1793734 | 45 | 0.3094462 |
| Sex | 3.3702996 | 3 | 0.3379775 |
| Therapy type: Group | 2.0443094 | 3 | 0.5632612 |
| Main diagnosis: GAD | 2.0336691 | 3 | 0.5654481 |
| Personality disorder score | 1.6103339 | 3 | 0.6570489 |
| Number of comorbidities | 1.4102917 | 3 | 0.7031241 |
| University degree | 1.3529473 | 3 | 0.7166002 |
| Ethnicity | 0.9901494 | 3 | 0.8036355 |
| Number of episodes | 0.9376265 | 3 | 0.8163395 |

## VIFS

**Title:** S.Table 11. Variance inflation factors estimated from the covariance matrix of parameter estimates, based on the correlation matrix from the information matrix (Harrell 2020). The maximum VIF calculated across all analyses was for age of onset (VIF=1.29, i.e. an inflation of 29%, which is moderate but not considered as problematic.

| Variable | VIF |
| --- | --- |
| Age of onset | 1.29 |
| Age during therapy | 1.28 |
| Personality disorder score | 1.19 |
| Number of episodes | 1.17 |
| Number of comorbidities | 1.11 |
| University degree | 1.06 |
| Sex | 1.05 |
| Regular therapeutic activity | 1.04 |
| Concurrent medications | 1.04 |
| First therapy | 1.03 |
| Therapy type | 1.02 |
| Ethnicity | 1.01 |

## Z-tests

**Title:** S.Table 12. Summary statistics from two-sample z-tests comparing effect sizes between the multivariable model (i.e. adjusted ORs) and univariable models (i.e. unadjusted ORs). Complete cases refers to a model in which individuals with missing data on any single analysis variable were dropped from the analysis, whereas in the main analysis missing data indicators were used to retain all individuals. Only statistically significant differences (p<0.05) are presented.

| **Variable** | **Model_1** | **Model_2** | **OR_1** | **OR_2** | **Z_score** | **P_value** |
| --- | --- | --- | --- | --- | --- | --- |
| Number of comorbidities | Multivariable | Univariable | 0.98 | 0.87 | 3.07 | 0.00214 |
| Age of onset | Multivariable | Univariable | 1.00 | 1.02 | -2.48 | 0.01310 |
| University degree (yes) | Multivariable | Univariable | 1.37 | 1.76 | -2.36 | 0.01830 |
| Number of episodes | Multivariable | Univariable | 0.94 | 0.91 | 2.05 | 0.04040 |
| Age of onset | Multivariable (complete cases) | Univariable (complete cases) | 0.99 | 1.02 | -3.20 | 0.00137 |
| Number of comorbidities | Multivariable (complete cases) | Univariable (complete cases) | 0.99 | 0.87 | 2.97 | 0.00298 |
| University degree (yes) | Multivariable (complete cases) | Univariable (complete cases) | 1.31 | 1.75 | -2.51 | 0.01210 |

**Title:** S.Table 13. Summary statistics from two-sample z-tests comparing effect sizes between the main analysis (where missing data indicators were used to retain all individuals) and complete case analyses (in which individuals with missing data on any single analysis variable were dropped from the analysis). Only differences with p<0.5 are presented as examples (none were significant at p<0.05).

| **Variable** | **Model_1** | **Model_2** | **OR_1** | **OR_2** | **Z_score** | **P_value** |
| --- | --- | --- | --- | --- | --- | --- |
| First therapy (yes) | Multivariable (complete cases) | Multivariable | 0.66 | 0.85 | -1.48 | 0.139 |
| Therapy type (group) | Multivariable (complete cases) | Multivariable | 1.01 | 0.78 | 1.11 | 0.267 |
| Age of onset | Multivariable (complete cases) | Multivariable | 0.99 | 1.00 | -1.07 | 0.285 |

**Title:** S.Table 14. Summary statistics from two-sample z-tests comparing effect sizes between the multivariable models (adjusted ORs) stratified timing of therapy relative to baseline GLAD sign-up. Only statistically significant differences (p<0.05) are presented.

| **Variable** | **Model_1** | **Analysis_1** | **Analysis_2** | **OR_1** | **OR_2** | **Z_score** | **P_value** |
| --- | --- | --- | --- | --- | --- | --- | --- |
| Concurrent medications (yes) | Multivariable | Prior | Post | 1.22 | 0.69 | 2.85 | 0.00437 |
| Concurrent medications (yes) | Multivariable | Concurrent | Post | 1.23 | 0.69 | 2.66 | 0.00781 |
| Main diagnosis (gad) | Multivariable | Prior | Post | 1.34 | 0.86 | 2.46 | 0.01390 |
| Concurrent medications (yes) | Multivariable | Full | Post | 0.98 | 0.69 | 2.30 | 0.02140 |

**Title:** S.Table 15. Summary statistics from two-sample z-tests comparing effect sizes between the multivariable models (adjusted ORs) stratified time since therapy at follow-up (Full=10y,15y,5y). Only differences with p<0.5 are presented as examples (none were significant at p<0.05).

| Variable | Model_1 | Analysis_1 | Analysis_2 | OR_1 | OR_2 | Z_score | P_value |
| --- | --- | --- | --- | --- | --- | --- | --- |
| Concurrent medications (yes) | Multivariable | 15y | 5y | 0.94 | 1.08 | -1.05 | 0.294 |
| Years since therapy at followup | Multivariable | 10y | 5y | 0.97 | 0.95 | 0.77 | 0.441 |
| Years since therapy at followup | Multivariable | 15y | 5y | 0.97 | 0.95 | 0.74 | 0.459 |
| Concurrent medications (yes) | Multivariable | 10y | 5y | 0.98 | 1.08 | -0.72 | 0.472 |

# Supplementary figures

## Participants

**Title:** S.Fig 1. Flowchart of GLAD study participants included in analyses


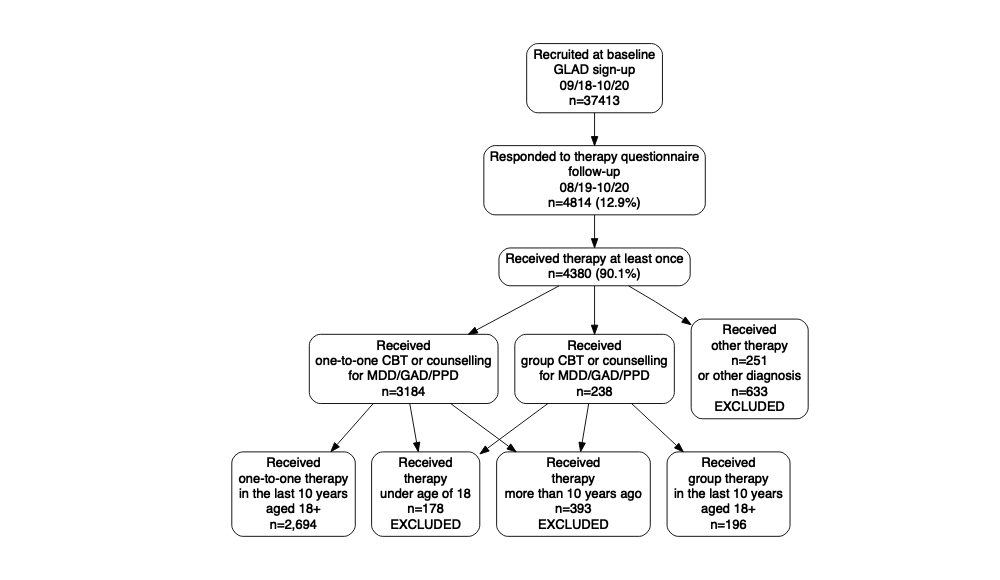


## Time-points

**Title:** S.Fig 2. Plots detailing time intervals between waves of data collection and receipt of psychological therapy


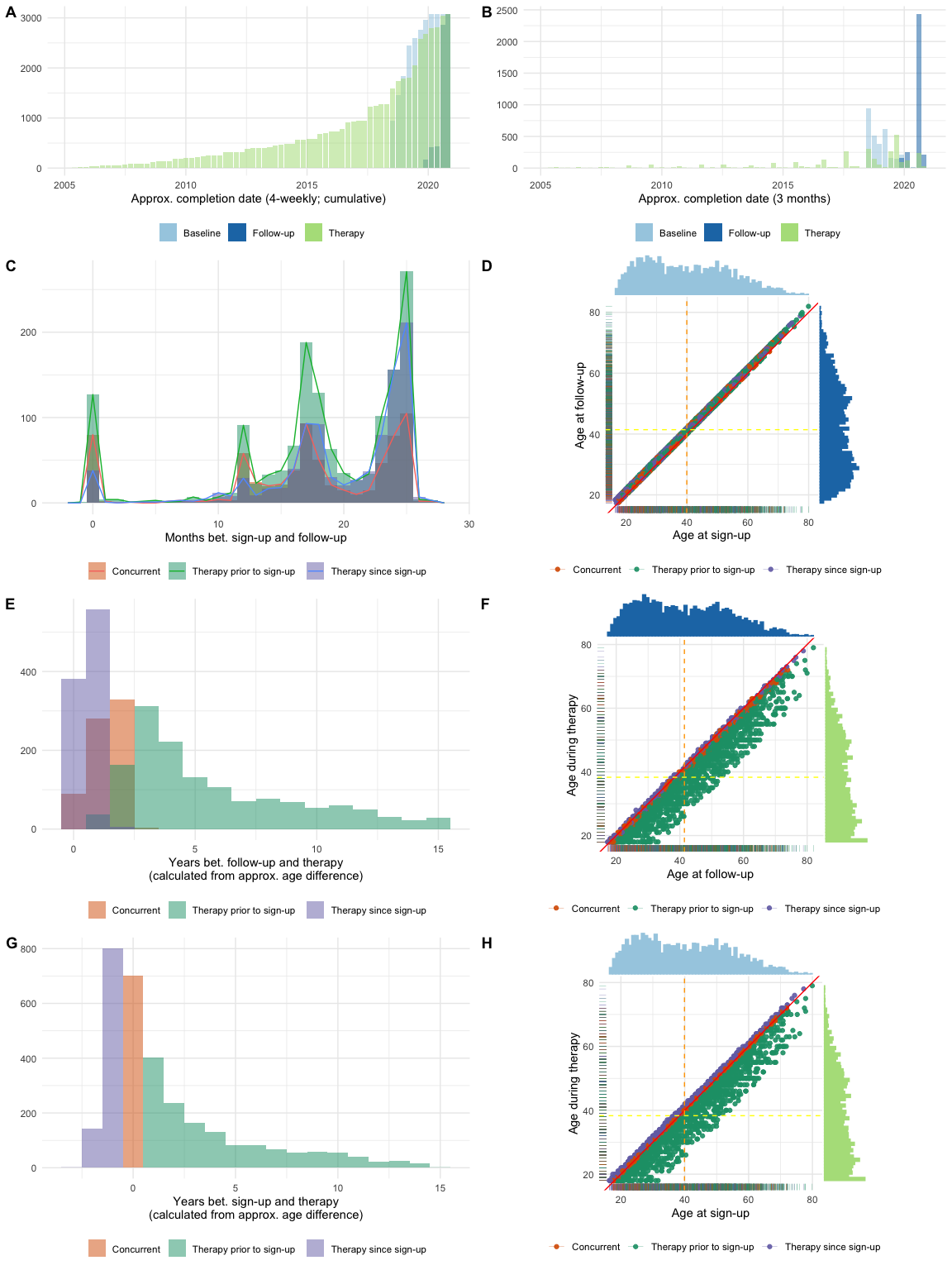


**Title:** S.Fig 3. Correlation matrix for all explanatory variables


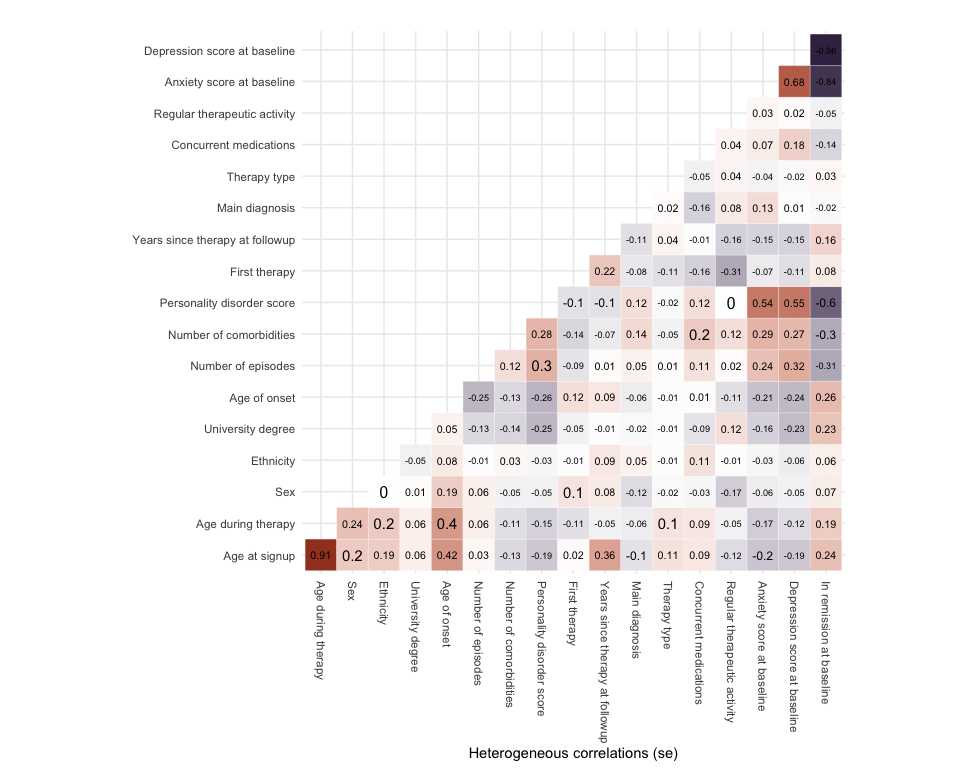


##

## Missing data

**Title:** S.Figure 3. Missing data indicators (yes/no) associated with retrospectively self-reported psychological therapy outcomes (global rating of improvement) in a subsample of the Genetic Links to Anxiety and Depression (GLAD) study participants (n=2890) who received psychological therapy (cognitive behavioural therapy or counseling) for major depressive disorder, generalised anxiety disorder, or phobic/panic disorders. **Legend:** Odds ratios, 95% confidence intervals and p values estimated from multivariable and univariable proportional odds ordinal logistic regression models using maximum likelihood estimation on retrospectively self-reported therapy outcome, measured via the global rating of change. Bonferroni-adjusted p value threshold was 0.0038 to correct for 13 effectively independent tests. *Note*: Reference category: No missing data on each variable; Effect of having missing data on each variable


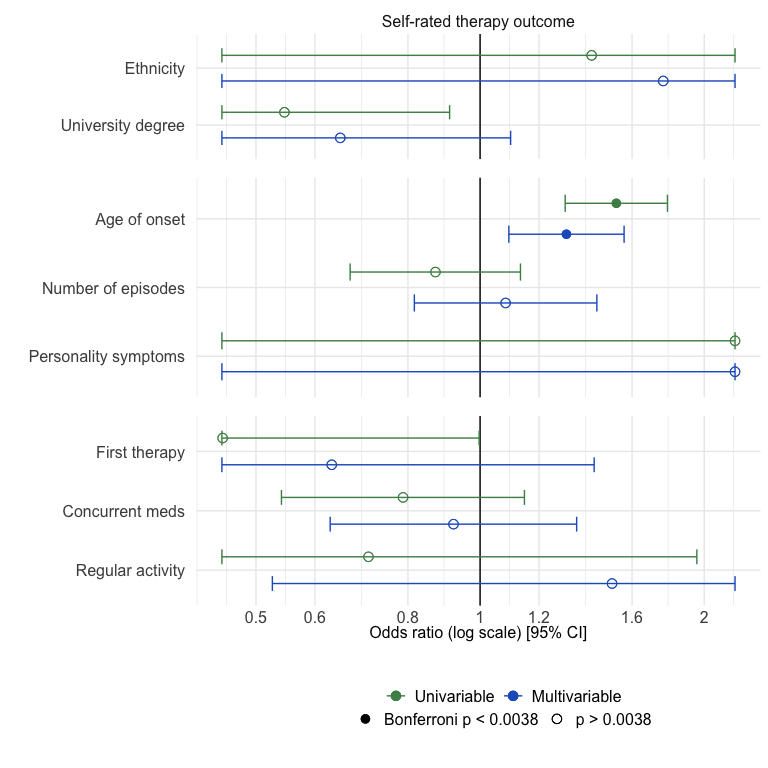


## Sensitivity analysis

### Time: therapy relative to sign-up

## Title: S.Figure 4. Summary statistics from multivariable (MV) proportional odds ordinal logistic regression models using maximum likelihood estimation to test for associations between self-rated therapy outcomes (global rating of improvement) and sociodemographic, clinical and therapy factors self-reported in a subsample of the Genetic Links to Anxiety and Depression (GLAD) study participants (n=2890) who received psychological therapy (cognitive behavioural therapy or counseling) for major depressive disorder, generalised anxiety disorder, or phobic/panic disorders. *Analyses were stratified by approximate timing of therapy relative to timing of the baseline sign up questionnaire.* Odds ratios, 95% confidence intervals and p-values.

## Legend: Order from left to right: 1. Full unstratified model (main analysis reported in manuscript - provided for easy comparison; n=2890); 2.“Prior” stratum - i.e. those who received their most recent course of therapy prior to signing up and completing the baseline questionnaire; n=944); 3. “Concurrent” stratum - i.e. those who were receiving their most recent course of therapy approximately concurrently to signing up and completing the baseline questionnaire; n=701); 4. “Post” stratum - i.e. those who received their most recent course of therapy after signing up and completing the baseline questionnaire; n=1437). *See S.Figure 2 for more details*

##
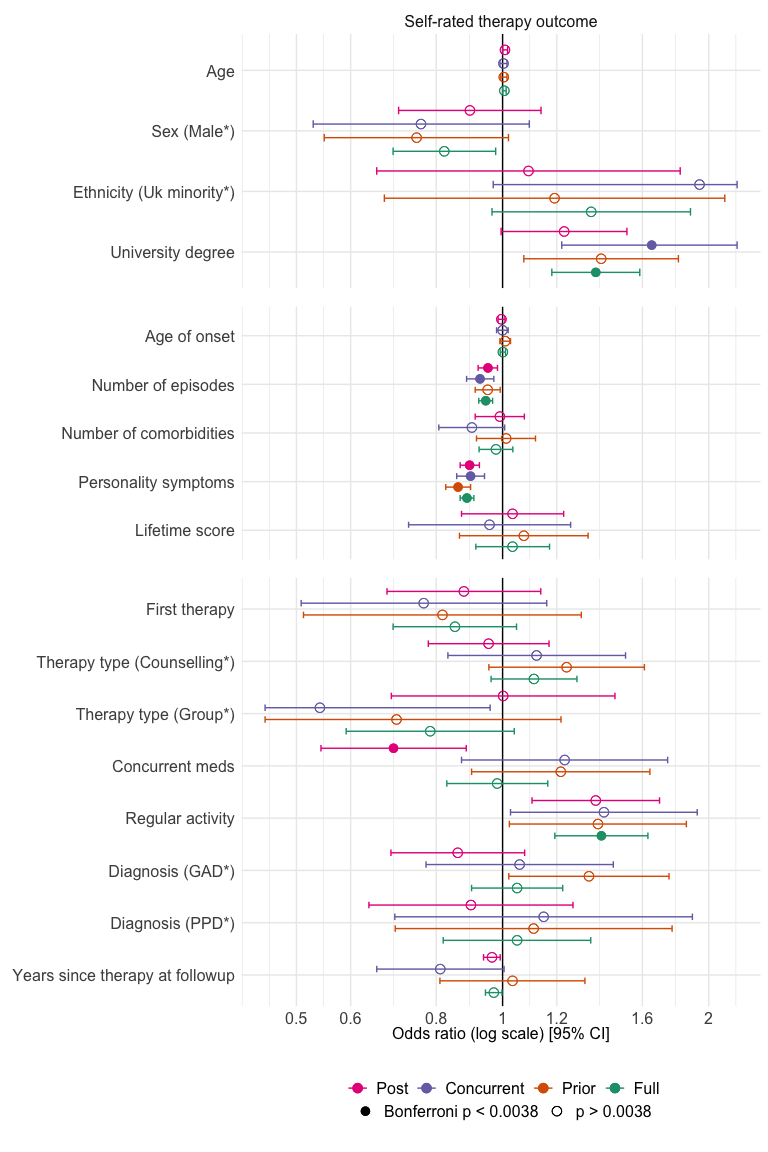


### Time: since therapy at time of follow-up

**Title:** S.Figure 5. Summary statistics from multivariable (MV) proportional odds ordinal logistic regression models using maximum likelihood estimation to test for associations between self-rated therapy outcomes (global rating of improvement) and sociodemographic, clinical and therapy factors self-reported in a subsample of the Genetic Links to Anxiety and Depression (GLAD) study participants (n=2890) who received psychological therapy (cognitive behavioural therapy or counseling) for major depressive disorder, generalised anxiety disorder, or phobic/panic disorders. *Threshold for inclusion in the main analysis was 10 years time difference between when the participant received therapy and when they completed the follow-up questionnaire (2010-2020). Sensitivity analyses were performed using two additional thresholds: 5 years (2015-2020) and 15 years (2005-2020).* Odds ratios, 95% confidence intervals and p-values

**Legend:** Odds ratios, 95% confidence intervals and p values estimated from multivariable proportional odds ordinal logistic regression models using maximum likelihood estimation on retrospectively self-reported therapy outcome, measured via the global rating of change. Bonferroni-adjusted p value threshold was 0.0038 to correct for 13 effectively independent tests. *Note*: *=Effect category. Reference categories: Sex Female); Ethnicity (white British); Diagnosis (Major Depressive Disorder); Therapy type (Cognitive Behavioural Therapy)


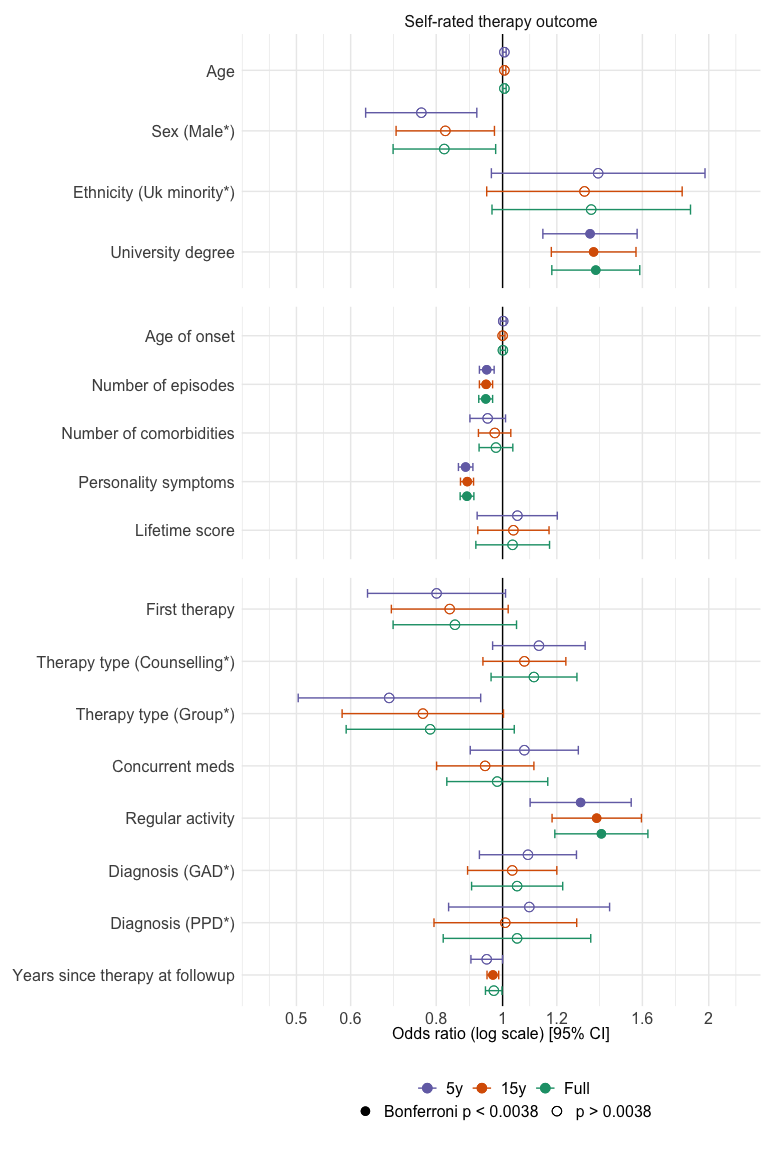


# Questionnaire

## Therapy history and outcomes questionnaire"

**Section 1 - Screening questions**

- Have you **ever** tried a talking therapy (e.g. one-to-one therapy or group therapy) or some other structured therapeutic activity (e.g. yoga, mindfulness, online self-help) for help with your anxiety or depression?
- Have you tried talking therapy (e.g. one-to-one therapy or group therapy) **more than once?** Please note: “more than once” refers to courses of treatment, rather than sessions of therapy.
- Have you tried **more than one** type of structured therapeutic activity? (e.g. yoga, mindfulness, online self-help)

## Correlations

**Section 2 - Most recent talking-therapy**

- For the most recent time you tried talking therapy, **how old were you** when you were receiving this therapy?
- What was the **main diagnosis** you were receiving this therapy for? (please only tick the main diagnosis)
  - Depression
  - Anxiety
  - Panic attacks
  - Fear or Phobia
  - Social anxiety
  - Other ……….
- Had you also received **additional diagnoses** from the doctor, during this time? (please tick all that apply)
  - Depression
  - Anxiety
  - Panic attacks
  - Fear or Phobia
  - Social anxiety
  - Other ……….
- For the most recent time you tried talking therapy, was this therapy **one-to-one therapy or group therapy?**
  - One-to-one
  - Group
  - Other ………
  - Don’t Know
- For the most recent time you tried talking therapy, **which type** of talking therapy did you try?
  - Cognitive behavioural therapy (CBT)
  - Counselling
  - Other ……….
  - Don’t Know
- **How much did your symptoms and day to day functioning improve after the most recent time you had talking therapy?**
  - Much better
  - A little better
  - No change
  - A little worse
  - Much worse
- For the most recent time you tried talking therapy, were you also **taking antidepressant or anti-anxiety medications**, during this course of psychological therapy?
  - No
  - Yes - Antidepressant medications
  - Yes - Anti-anxiety medications
  - Yes - Both

**Section 3 - Second most recent talking-therapy** (same format as section 2)

**Section 4 - Structured therapeutic activity 1**

- Which **type of structured therapeutic activity** have you **used most** to help with your anxiety or depression? (please select the one activity that you have used most)
  - Well-being workshop
  - Mindfulness class
  - Face-to-face support group
  - Online e-learning package or guided self-help
  - Online support group
  - Mindfulness or guided meditation app
  - Self-help book
  - Yoga
  - Physical exercise programme
  - Other: ……………………………
  - Don’t know
- How much did your symptoms and day to day functioning **improve** after using this structured therapeutic activity?
  - Much better
  - A little better
  - No change
  - A little worse
  - Much worse
- What **symptoms** were you using the activity to help with? (please tick all that apply)
  - Depression
  - Anxiety
  - Panic attacks
  - Fear or Phobia
  - Social anxiety
  - Other ……………………………
- Were you also taking anti-depressant or anti-anxiety **medications** during the course of this activity?
  - No
  - Yes - Antidepressant medications
  - Yes - Anti-anxiety medications
  - Yes - Both

**Section 5 - Structured therapeutic activity 1** (same format as section 4)

- Which **other** type of structured therapeutic activity have you also used to help with your anxiety or depression? (please select one other activity that you have used frequently)
